# Artemisinin versus Nonartemisinin Combination Therapy for Uncomplicated Malaria: Randomized Clinical Trials from Four Sites in Uganda

**DOI:** 10.1371/journal.pmed.0020190

**Published:** 2005-07-26

**Authors:** Adoke Yeka, Kristin Banek, Nathan Bakyaita, Sarah G Staedke, Moses R Kamya, Ambrose Talisuna, Fred Kironde, Samuel L Nsobya, Albert Kilian, Madeline Slater, Arthur Reingold, Philip J Rosenthal, Fred Wabwire-Mangen, Grant Dorsey

**Affiliations:** 1 Ministry of Health, Kampala, Uganda,; **2** Department of Medicine, San Francisco General Hospital, University of California, San Francisco, California, United States of America,; **3** Makerere University Medical School, Kampala, Uganda,; **4** Division of Epidemiology, School of Public Health, University of California, Berkeley, California, United States of America,; **5** Institute of Public Health, Makerere University, Kampala, Uganda; Mahidol UniversityThailand

## Abstract

**Background:**

Drug resistance in *Plasmodium falciparum* poses a major threat to malaria control. Combination antimalarial therapy including artemisinins has been advocated recently to improve efficacy and limit the spread of resistance, but artemisinins are expensive and relatively untested in highly endemic areas. We compared artemisinin-based and other combination therapies in four districts in Uganda with varying transmission intensity.

**Methods and Findings:**

We enrolled 2,160 patients aged 6 mo or greater with uncomplicated falciparum malaria. Patients were randomized to receive chloroquine (CQ) + sulfadoxine-pyrimethamine (SP); amodiaquine (AQ) + SP; or AQ + artesunate (AS). Primary endpoints were the 28-d risks of parasitological failure either unadjusted or adjusted by genotyping to distinguish recrudescence from new infections.

A total of 2,081 patients completed follow-up, of which 1,749 (84%) were under the age of 5 y. The risk of recrudescence after treatment with CQ + SP was high, ranging from 22% to 46% at the four sites. This risk was significantly lower (*p* < 0.01) after AQ + SP or AQ + AS (7%–18% and 4%–12%, respectively). Compared to AQ + SP, AQ + AS was associated with a lower risk of recrudescence but a higher risk of new infection. The overall risk of repeat therapy due to any recurrent infection (recrudescence or new infection) was similar at two sites and significantly higher for AQ + AS at the two highest transmission sites (risk differences = 15% and 16%, *p*< 0.003).

**Conclusion:**

AQ + AS was the most efficacious regimen for preventing recrudescence, but this benefit was outweighed by an increased risk of new infection. Considering all recurrent infections, the efficacy of AQ + SP was at least as efficacious at all sites and superior to AQ + AS at the highest transmission sites. The high endemicity of malaria in Africa may impact on the efficacy of artemisinin-based combination therapy.

The registration number for this trial is ISRCTN67520427 (http://www.controlled-trials.com/isrctn/trial/|/0/67520427.html).

## Abstract 2

Artemisinin-based combination therapy for malaria may be effective at treating primary infection, but may not be the best option in highly endemic areas because of risk of recurrence.

## Introduction

Malaria remains one of the most serious global health problems and a leading cause of childhood morbidity and mortality, especially in Africa [1]. Efforts to control malaria in Africa have been severely compromised by the emergence of resistance in *Plasmodium falciparum* to the inexpensive and widely used drugs, chloroquine (CQ) and sulfadoxine-pyrimethamine (SP) [2,3]. Use of combination antimalarial therapy, particularly newer regimens containing artemisinin-based compounds, has been widely advocated [4]. However, concerns regarding the cost and availability of artemisinin-based combination therapy (ACT) remain, and limited data comparing ACT with other combination therapies in Africa are available [5]

In 2000, Uganda replaced CQ with the combination of CQ + SP as the recommended first-line treatment for uncomplicated malaria, although efficacy data for this regimen were lacking. To support development of a rational antimalarial treatment policy, the Uganda Malaria Surveillance Project was formed as a collaborative effort between the Ugandan Malaria Control Program, the East African Network for Monitoring Antimalarial Therapy, and academic researchers to provide efficacy data on antimalarial therapies from multiple sites. In addition to providing efficacy data from diverse locales, this project offers the possibility of evaluating the impacts of varied malaria endemicities and drug resistance patterns on responses to antimalarial therapy.

In a recent study from a relatively low-transmission site in Kampala, Uganda, combinations of amodiaquine (AQ) + SP and AQ + artesunate (AS) were found to be significantly more efficacious than CQ + SP. Directly comparing the two AQ-containing regimens over 28 d, AQ + SP was associated with a higher risk of recrudescence after therapy, and AQ + AS was associated with a higher risk of new infections, such that the overall risk of repeat therapy was similar [6]. Considering all recurrent infections, the ACT regimen did not offer an obvious advantage over the much less expensive AQ + SP combination. However, this conclusion led to concerns that results may have been unique to our study site and that the implications of recrudescent and new infections might be very different [7]. Here we report the results of randomized clinical trials comparing CQ + SP, AQ + SP, and AQ + AS at four additional sites in Uganda, conducted with the aim of providing data from areas of varying transmission intensity to help guide antimalarial treatment policy. Furthermore, we were interested in evaluating the interplay between transmission intensity and the efficacies of different combination regimens, considering effects both on recrudescences and new infections after therapy.

## Methods

### Study Design and Study Sites

**Figure 1:**
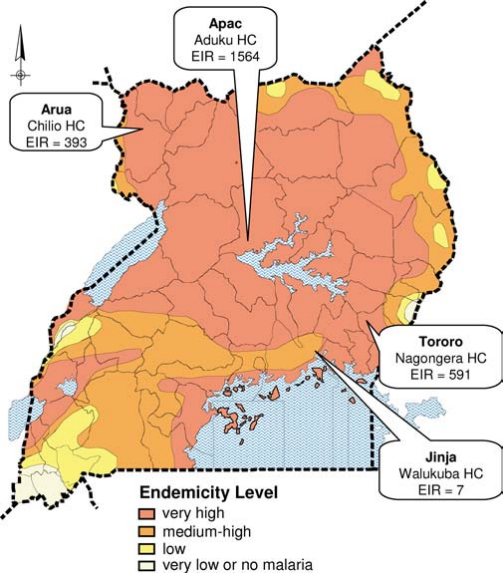
Map of Uganda Based on Malaria Endemicity Very low or no malaria: unstable malaria with varying parasite rates in children but generally below 5% (altitude above 1,700 m); low: parasite rates in children below 10% (altitude 1,500–1,700 m); medium to high: parasite rates in children above 10% and generally below 50%, except for seasonal peaks (altitude 1,300–1,500 m); very high: parasite rates in children above 50% (altitude below 1,300 m) (Malaria Control Program, Uganda Ministry of Health). EIR, entomological inoculation rate.

We conducted single-blind, randomized clinical trials to compare the efficacy and safety of three combination antimalarial regimens at four district health centers, using a common study protocol. Study sites, selected for geographic diversity, were: Jinja (periurban, southern Uganda, medium-high endemicity), Arua (rural, northwest Uganda, very high endemicity), Apac (rural, central Uganda, very high endemicity), and Tororo (rural, southeast Uganda, very high endemicity) (Figure 1). The level of transmission intensity was further characterized based on recent estimates of entomological inoculation rates (the number of infective bites per person per year): Jinja = 7, Arua = 393, Tororo = 591, Apac = 1,564 (P. Okello, Uganda Ministry of Health, personal communication). The study protocol was approved by the Uganda National Council of Science and Technology and the institutional review boards of the University of California, San Francisco and the University of California, Berkeley (Protocols S1 and S2).

### Patients

Consecutive patients presenting to district health centers between November 2002 and May 2004 with symptoms suggestive of malaria and a positive-screening thick blood smear were referred to study physicians. Patients were enrolled if they fulfilled the following selection criteria: (1) age 6 mo or greater; (2) history of fever in the last 24 h or axillary temperature > 37.5 °C; (3) no history of serious side effects to study medications; (4) no evidence of a concomitant febrile illness; (5) provision of informed consent; (6) no history of treatment with an antifolate or AQ during the previous week; (7) absence of pregnancy-based on history of last menstrual period; (8) no danger signs or evidence of severe malaria [8]; and (9) *P. falciparum* monoinfection with parasite density 2,000/μl–200,000/μl. Because laboratory results were generally not available until the following day, a patient could be excluded after randomization.

### Treatment, Randomization, and Blinding

Patients were randomly assigned to receive one of three oral therapies: CQ (10 mg/kg on days 0 and 1, and 5 mg/kg on day 2) + SP (25 mg/kg sulfadoxine and 1.25 mg/kg pyrimethamine as a single dose on day 0); AQ (10 mg/kg on days 0 and 1, and 5 mg/kg on day 2) + SP; or AQ + AS (4 mg/kg on days 0, 1, and 2). Patients in the CQ + SP and AQ + SP treatment arms also received lactose placebo tablets on days 1 and 2.

Randomization codes were computer-generated by an off-site investigator for two age groups (6–59 mo and 5 y or older) and provided to a study nurse responsible for treatment allocation. All other study personnel were blinded to the treatment assignments, and patients were not informed of their treatment regimen. Patients were directly observed for 30 min after treatment, and the dose was readministered if vomiting occurred. Patients who repeatedly vomited their first dose of study medication were excluded from the study.

### Follow-Up Procedures and Classification of Treatment Outcomes

Following enrollment, patients were asked to return for follow-up visits on days 1, 2, 3, 7, 14, 21, 28, and any other day when they felt ill. Blood was obtained by finger prick for thick blood smears and storage on filter paper on all follow-up days except day 1. Hemoglobin measurements were repeated on day 28 or the day of treatment failure.

Treatment outcomes were classified according to 2003 World Health Organization guidelines as early treatment failure (ETF; danger signs or complicated malaria or failure to adequately respond to therapy days 0–3), late clinical failure (LCF; danger signs or complicated malaria or fever and parasitemia on days 4–28 without previously meeting criteria for ETF), late parasitological failure (LPF; asymptomatic parasitemia on day 28 without previously meeting criteria for ETF or LCF), and adequate clinical and parasitological response (ACPR; absence of parasitemia on day 28 without previously meeting criteria for ETF, LCF, or LPF) [8]. Patients classified as treatment failures were treated with quinine (10 mg/kg three times daily for 7 d); however, their response to repeat therapy was not assessed. Patients were excluded after enrollment if any of the following occurred: (1) use of antimalarial drugs outside of the study protocol; (2) parasitemia in the presence of a concomitant febrile illness; (3) withdrawal of consent; (4) loss to follow-up; (5) protocol violation; and (6) death due to a nonmalaria illness.

### Laboratory Procedures

Blood smears were stained with 2% Giemsa for 30 min. Parasite densities were determined from thick blood smears by counting the number of asexual parasites per 200 WBCs (or per 500, if the count was less than 10 parasites/200 WBCs), assuming a WBC count of 8,000/μl. A smear was considered negative if no parasites were seen after review of 100 high-powered fields. Thin blood smears were used to detect nonfalciparum infections. Hemoglobin measurements were made using a portable spectrophotometer (HemoCue, Anglholm, Sweden). Molecular genotyping techniques were used to distinguish recrudescence from new infection for all patients failing therapy after day 3. Briefly, filter paper blood samples collected on the day of enrollment and the day of failure were analyzed for polymorphisms in merozoite surface protein-2 (MSP-2) using nested-PCR as previously described [9]. Genotyping patterns on the day of repeat therapy were compared with those at treatment initiation using GelCompar II software (Applied Maths, St-Martens-Latem, Belgium). An outcome was defined as recrudescence if all alleles present at the time of retreatment were present at the time of treatment initiation, and as a new infection otherwise.

### Statistical Analysis

The study was designed to test the hypothesis that treatment with AQ + SP or AQ + AS would change the risk of recrudescence compared to CQ + SP at each study site. We calculated that 176 patients were needed to be enrolled in each treatment arm at each site for a 0.05 two-sided type I error with 0.8 power, assuming a risk of recrudescence adjusted by genotyping of 15% in the CQ + SP group and 5% in the comparison groups.

Data were double-entered and verified using EpiInfo 6**.**04 (Centers for Disease Control and Prevention, Atlanta, Georgia, United States), and analyzed using Stata version 8.0 (Stata Corporation, College Station, Texas, United States). Efficacy data were evaluated using a per-protocol analysis, which only included patients with treatment outcomes. Prior to completion of this study we decided that a per-protocol analysis would be more appropriate than an intention-to-treat (ITT) analysis. Our protocol was used to study CQ + SP versus AQ + SP at three additional sites prior to the completion of the studies presented here. During the analysis of these previous studies we decided that a per-protocol analysis provided better estimates of the true risk of treatment outcomes than an ITT analysis, as in an ITT analysis one must assign treatment outcomes to patients who did not complete the study. In addition, there were so few patients enrolled who did not complete the study at all our sites that the comparative results using a per-protocol analysis did not differ from that using an ITT analysis. Primary efficacy outcomes included the 28-d risks for all recurrent infection (ETF, LCF, or LPF), recrudescence (all ETF and any LCF or LPF categorized as recrudescence based on genotyping results), and new infections (any LCF or LPF categorized as new infection based on genotyping results). Risks of recrudescence and new infection were estimated using the Kaplan-Meier product limit formula with censoring for the competing risk (new infections censored when estimating risks of recrudescence and vice versa). Secondary outcomes included the risk of recurrent infection unadjusted by genotyping at day 14, presence of fever on days 1–3, parasitemia on days 2 and 3, change in hemoglobin level between the day of enrollment and the last day of follow-up, presence of gametocytes during any follow-up day, and the incidence of adverse events. Pairwise comparisons of treatment efficacy were made using risk differences with exact 95% confidence intervals (CIs). Other categorical variables were compared using chi-squared or Fisher's exact test, and continuous variables were compared using the independent samples *t*-test. All reported *p*-values were two-sided, without adjustment for multiple testing, and were considered statistically significant if less than 0.05.

## Results

### Enrollment

**Figure 2:**
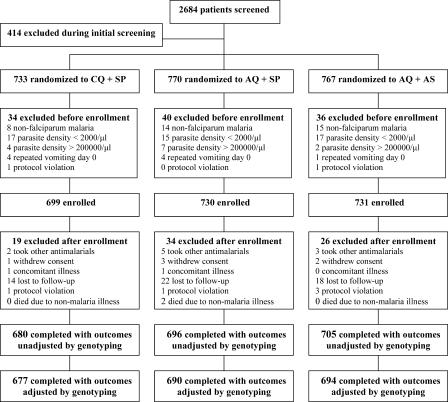
Trial Profile Trial profile stratified by treatment group and site (Jinja, Arua, Tororo, Apac); screened: (693, 625, 718, 648); excluded during initial screening: (122, 74, 157, 61); randomized: CQ+SP (179, 184, 174, 196); AQ + SP (196, 185, 192, 197); AQ + AS (196, 182, 195, 194); excluded before enrollment: CQ + SP (4, 11, 8, 11); AQ + SP (5, 14, 11, 10); AQ + AS (8, 20, 1, 7); enrolled: CQ + SP (168, 180, 166, 185); AQ + SP (186, 180, 181, 183); AQ + AS (189, 174, 194, 174); excluded after enrollment: CQ + SP (8, 2, 4, 5); AQ + SP (13, 7, 9, 5); AQ + AS (8, 3, 13, 2); completed: CQ + SP (160, 178, 162, 180); AQ + SP (173, 173, 172, 178); AQ+AS (181, 171, 181, 172).

Of 2,684 patients who underwent screening, 2,270 were randomized to treatment, and 2,160 were enrolled in the studies (Figure 2). Primary efficacy outcomes, unadjusted and adjusted by genotyping, were available for 96% and 95% of patients enrolled, respectively.

### Baseline Characteristics

Baseline characteristics of the patients across the three treatment groups were similar at each site (Table 1). Between the four sites, patients differed in some baseline characteristics, as expected based on differences in transmission intensity (Table 1). Patients from Jinja were older, with 39% age 5 y or older, compared to less than 10% at the other sites (*p* < 0.001), consistent with more common presentation of older individuals with malaria at a lower transmission site. Patients from Jinja also had higher baseline temperatures (*p* < 0.001) and mean hemoglobin levels (*p* < 0.001) compared to the other sites. Parasite densities varied significantly across the sites (*p* < 0.001 for all pairwise comparisons) and decreased with increasing entomological inoculation rate estimates. The proportion of patients with gametocytes present in pretreatment samples varied considerably (*p* < 0.001), ranging from 10% (Jinja and Tororo) to 51% (Apac). The proportion of patients providing a history of recent CQ use ranged from 5% (Arua) to 28% (Jinja), with 90% of patients reporting a partial treatment course (less than three doses) prior to presentation at the district health centers.

**Table 1:**
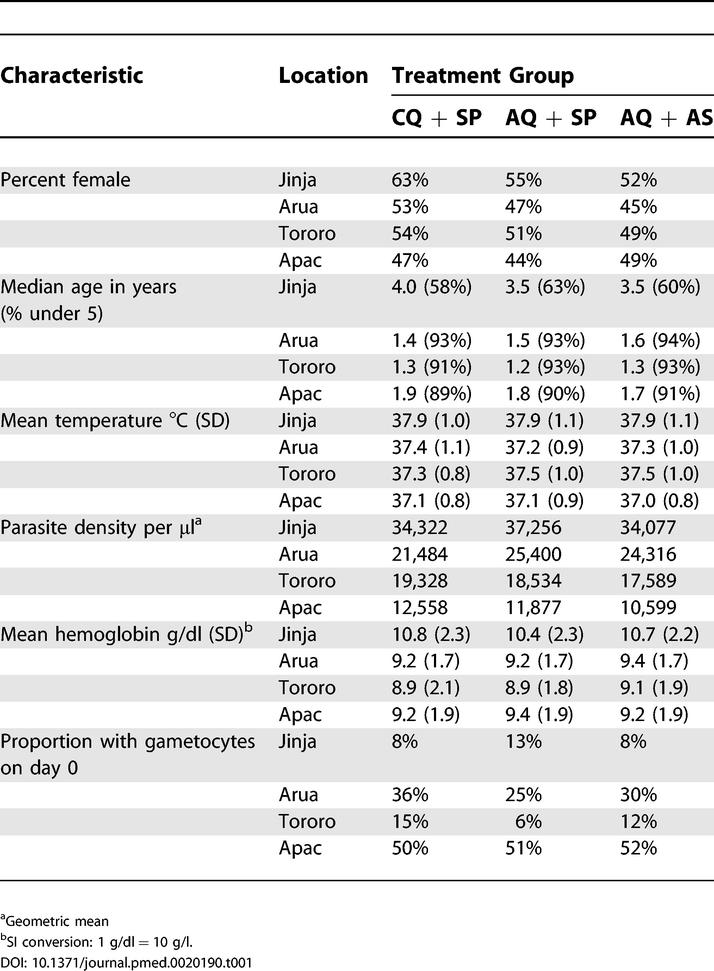
Baseline Characteristics of Patients Completing the Study

### Primary Outcome: Treatment Efficacy

ETFs were uncommon, but significant differences between the three treatment groups were observed when data from all sites were combined (CQ + SP = 3.4%, AQ + SP = 1.0%, AQ + AS = 0.1%; *p* < 0.04 for all pairwise comparisons). The risk of recurrent infection (unadjusted by genotyping) at day 14 was significantly higher for CQ + SP at all four sites (16%–57%) compared to AQ + SP (1%–11%) or AQ + AS (5%–13%) (Table 2). The risk of recurrent infection at day 28 was extremely high for CQ + SP at all four sites, ranging from 63% to 88% (Table 2). After adjustment by genotyping, the risk of recrudescence remained very high for CQ + SP at all four sites, ranging from 22%–46% (Table 2). Compared to CQ + SP, the risk of recurrent infection and recrudescence at day 28 was significantly lower for the AQ + SP (28%–59% and 7%–18%, respectively) and AQ + AS (19%–74% and 4%–12%, respectively) treatment groups (all *p*s < 0.05, Table 2).

**Table 2:**
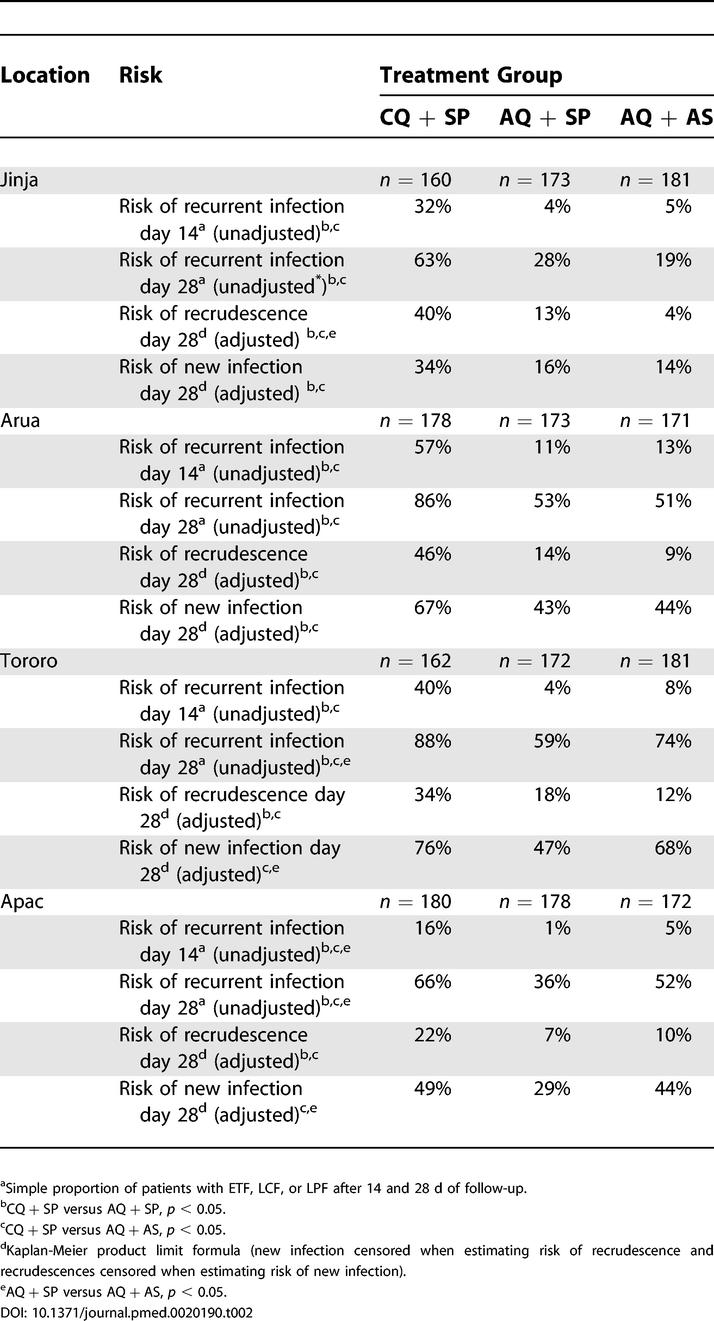
Primary Treatment Efficacy Outcomes

**Figure 3:**
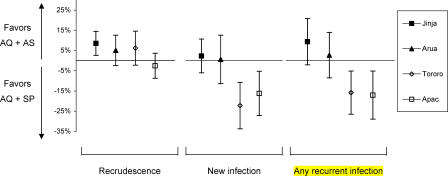
Comparison of AQ + SP versus AQ + AS Risk differences and 95% CIs for recrudescence (adjusted by genotyping), new infections (adjusted by genotyping), and any recurrent infection (unadjusted, recrudescence or new infection) at day 28.

The most interesting comparisons of treatment efficacy were seen with AQ + SP versus AQ + AS. AQ + SP was associated with a higher risk of recrudescence at three sites, although this reached statistical significance at only one site, Jinja (risk difference = 9%; 95% CI: 3%–15%; *p* = 0.009) (Figure 3). In contrast, the risk of new infection associated with AQ + SP and AQ + AS was similar at two sites, and was significantly higher with AQ + AS at the two highest transmission intensity sites, Tororo (risk difference = 21%; 95% CI: 10%–31%; *p* < 0.001) and Apac (risk difference = 15%; 95% CI: 5%–25%; *p* = 0.003) (Figure 3). Overall, the risk of any recurrent infection (recrudescence or new infection) was similar at the two lower transmission intensity sites and significantly higher with AQ + AS at the two highest transmission intensity sites; Tororo (risk difference = 15%; 95% CI: 5%–25%; *p* = 0.005) and Apac (risk difference = 16%; 95% CI: 6%–26%; *p* = 0.004) (Figure 3).

Although some patients classified as failures had asymptomatic parasitemia on day 28 (LPF), the majority (67%) were symptomatic on the day of failure (ETF or LCF). Comparisons of treatment efficacy (both unadjusted and adjusted by genotyping) considering only those patients who were symptomatic on the day of failure were similar to those above (data not shown).

Comparisons of the characteristics of late clinical and parasitological failures due to recrudescence versus those due to new infections are presented in Table 3. Median duration to failure was shorter with recrudescences compared to new infections (26 d versus 27 d, *p* = 0.03), although the difference was marginal, and over 75% of both recrudescences and new infections occurred after 20 d of follow-up. No differences between recrudescences and new infections were found with respect to the proportion of patients who were symptomatic, the risk of complicated malaria, parasite density, or changes in hemoglobin. Gametocytes during follow-up were common with both outcomes, but more common with recrudescences (51% versus 43%, *p* = 0.02).

**Table 3:**
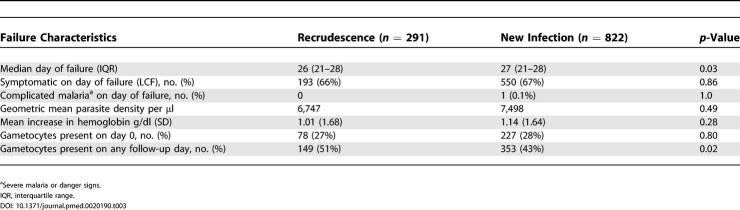
Comparison of Late Clinical and Parasitological Failures due to Recrudescence versus New Infection

### Secondary Outcomes

Treatment with AQ + AS was associated with a significantly lower risk of parasitemia on day 2 at all four sites (2%–9%) compared to CQ + SP (37%–74%) or AQ + SP (33%–58%); however, this was not consistently associated with clinical benefit as measured by presence of fever and hemoglobin level (Table 4). The proportion of patients with temperature > 37.5 °C on day 2 was significantly lower in the AQ + AS group compared to CQ + SP at two sites, but there were no significant differences compared to the AQ + SP group (Table 4). Similar results were seen with temperature on days 1 and 3 and when considering subjective fever (data not shown). Increase in hemoglobin during follow-up was greatest in the AQ + SP group at all sites, reaching statistical significance at three sites when compared to CQ + SP and at one site when compared to AQ + AS (Table 4). The proportion of patients with gametocytes during follow-up was lowest in the AQ + AS group at all sites, reaching statistical significance at two sites when compared to CQ + SP and at one site when compared to AQ + SP (Table 4). When restricting the analysis to only those patients with no gametocytes on day 0, a similar trend was found, favoring AQ + AS, with statistical significance reached at all four sites when compared to CQ + SP and at two sites when compared to AQ + SP (Table 4).

**Table 4:**
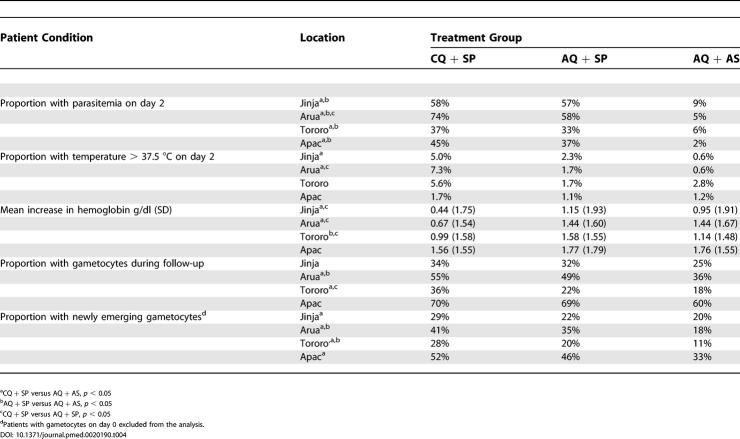
Secondary Treatment Outcomes

### Safety and Tolerability

Among 2,160 patients enrolled in the studies, 20 serious adverse events were reported in 16 patients (four CQ + SP, eight AQ + SP, four AQ + AS, *p* = 0.40). Serious adverse events included anemia (two AQ + SP, one AQ + AS), convulsion (one CQ + SP, two AQ + SP, one AQ + AS), dehydration (one AQ + AS), edema (one AQ + SP), malnutrition (one CQ + SP), mental status change (two AQ + SP), respiratory illness (one CQ + SP, two AQ + SP, one AQ + AS), vomiting (one CQ + SP), and weakness (one AQ + SP). One patient died of suspected severe malnutrition and one patient died of congestive heart failure due to a presumed congenital heart defect (both patients had received AQ + SP). All serious adverse events were deemed to be unlikely (ten events) or possibly (ten events) related to the study medications.

## Discussion

Antimalarial drug resistance is one of the greatest threats to malaria control in Africa [10]. In response to widespread resistance to CQ and SP, use of combination antimalarial therapy, particularly ACT, has been strongly advocated [11]. This study compared the efficacy of three different combination therapies, CQ + SP, currently the recommended first-line regimen in Uganda; AQ + SP, an inexpensive regimen that has proven to be efficacious in recent studies; and AQ + AS, an ACT regimen. More important, evaluations were made across differing levels of transmission intensity, allowing us to assess the impact of endemicity on treatment responses. We found that CQ + SP was highly ineffective. Indeed, data from various parts of Uganda have shown that the *pfcrt* K76T mutation primarily responsible for CQ resistance is virtually ubiquitous [12,13] and that the efficacy of CQ + SP is similar to that of SP alone [14]. Replacing CQ with AQ in combination with SP greatly reduced the risk of recrudescence and prevented new infections, a benefit which was most evident at the two highest transmission sites. The prevention of new infections by AQ + SP is likely due to the long elimination half-lives of both drugs [15,16]. Compared to AQ + SP, treatment with AQ + AS was generally associated with a lower risk of recrudescence but a higher risk of new infection within the month after therapy. This is likely due to the fact that AS is rapidly eliminated, leaving only AQ to provide posttreatment prophylaxis. When comparing AQ + AS to AQ + SP, the overall risks of recurrent infection were similar at two study sites and significantly higher for AQ + AS at the two highest transmission sites.

Differences in treatment efficacies between our study sites could not be compared formally, as patient populations differed between sites. Nonetheless, wide differences in efficacies between sites were seen. These differences may have been due both to variations in endemicity (and therefore antimalarial immunity) and to varied drug resistance patterns. Varied endemicity likely played an important role, as suggested by marked differences in baseline characteristics between the study populations. Considering varied drug resistance patterns, preliminary data analyzing parasites from the four sites suggest that the prevalences of mutations known to mediate resistance to CQ and SP were similar (unpublished data). Thus, the major factor mediating differences in outcomes between study sites appears to be differences in the antimalarial immunity of study populations.

Antimalarial drug-efficacy studies that limit follow-up to 14 d or less may significantly underestimate the risk of recrudescence [17]. Our study shows that this underestimation is particularly problematic in regions with very high malarial endemicity. Overall, only 21% of recrudescences occurred within the first 2 wk of the 4-wk follow-up period, and this proportion decreased to 14% at the highest transmission site. Thus, in highly endemic areas recrudescence may be delayed, presumably due to the contribution of host immunity on initial parasite clearance, and this delay appears to increase with increasing transmission intensity [17]. Additionally, the risk of new infections can be substantial in highly endemic areas. Indeed, in our study 72% of all recurrent infections were due to new infections, and with our two most efficacious regimens (AQ + SP and AQ + AS) this proportion was 80%. Although comparisons of antimalarial drug efficacy generally do not consider the relative impact on the risk of new infections, this factor can play a large role in treatment outcomes, especially in high-transmission areas such as Africa. Our identification of frequent new infections emphasizes the importance of other malaria control measures, such as the use of bed nets, vector reduction, and possibly intermittent presumptive therapy, as ways of reducing the risk of new infections and maximizing the impact of therapy.

Follow-up in this study was limited to 28 d. In a previous longitudinal study of SP alone or in combination with AQ or AS from Uganda, 72% of recrudescences occurred within 28 d and 81% within 42 d [18]. Thus, the overall risk of recrudescence in this study was likely underestimated. Similarly, we may have underestimated the impacts of the different therapies on risks of new infection. These limitations will be present in any study with distinct endpoints for efficacy assessment, but it is generally agreed that 28-d outcomes offer a reasonable comparative assessment of antimalarial therapies [8]. However, the best comparisons of the impacts of different therapies on the overall risk of repeat therapy can only be made using a longitudinal study design with extended follow-up covering multiple episodes of malaria, as we described previously [18].

We suggest that antimalarial therapies be judged not only by their impact on the risk of recrudescence, but also by their impact on the risk of new infections after therapy [6]. However, it has been argued that the consequences of recrudescence and new infection are not equal and that patients who have recrudescence face a greater risk of progression to complicated malaria and death [7]. Our data do not support this contention. In our study, the risk of ETF was remarkably low (<2%), and only four of 2,081 patients progressed to severe malaria or danger signs requiring therapy with intravenous quinine during the first 3 d of follow-up. Among 1,133 failures identified 4–28 d after therapy, 760 (67%) patients were symptomatic (LCFs), and clinical differences between recrudescence and new infection were not apparent. The only episode of severe malaria/danger signs to occur after day 3 was caused by a new infection. Thus, we found no support for the argument that the clinical consequences of recrudescence after treatment are different from those of a new infection during our 28 d follow-up period. It has also been argued that recrudescences are more difficult to treat than new infections. We could not address this issue in this study as patients were not assessed following repeat therapy. However, treatment of recrudescent infections was compared to initial treatment in a previous longitudinal study. Although the treatment of recrudescent infections with SP was associated with a higher rate of treatment failure, interestingly, treatment with SP + AS was associated with a lower rate of treatment failure [19]. Additional studies with longer follow-up, covering repeated episodes of malaria, are needed to more fully evaluate the treatment implications of recrudescent infections.

The classification of recrudescence and new infection depends on the genotyping methods used. In our study we used a highly specific definition of recrudescence that required all alleles present at the time of retreatment be present at the time of treatment initiation. As recurrent infections containing both new alleles and alleles present at the treatment initiation were classified as new infections, we may have underestimated the true risk of recrudescence. Defining recrudescence as having any allele at the time of retreatment present at the time of treatment initiation increased our point estimates of the risk of recrudescence. However, using this more sensitive (but less specific) definition of recrudescence did not have a significant impact on comparative efficacies. Indeed, we suggest that the most useful comparisons of the clinical consequences of different treatments should be based on treatment outcome results unadjusted by genotyping, including all retreatments for clinical illness.

Artemisinin derivatives are highly attractive antimalarials because they act rapidly, are well tolerated, and are currently not limited by resistance [3]. However, artemisinin monotherapy is associated with a high risk of recrudescence, necessitating use of artemisinins in combination with other antimalarials to achieve maximum efficacy [20]. In Thailand, the combination of AS + mefloquine has been associated with sustained cure rates over 95% and a decreased incidence of malaria [21,22]. The coformulated ACT, artemether-lumefantrine, has been shown to be highly efficacious in relatively low endemicity sites in Southeast Asia [23–25]. Published evidence for the effectiveness of ACT in highly endemic areas of Africa remains limited. The combination of CQ + AS was associated with a 47%–93% risk of treatment failure (unadjusted by genotyping) after 28 d in three West African countries [26]. SP + AS was associated with a higher risk of treatment failure (unadjusted and adjusted by genotyping) compared to AQ + SP in Uganda and Rwanda [18,27]. AQ + AS was associated with a lower risk of recrudescence but a similar risk of overall treatment failure when compared to AQ + SP at a site with relatively low-transmission intensity in Uganda [6].

Although ACT is clearly better than standard monotherapies, the somewhat disappointing results of ACT in Africa probably relate to the high endemicity of malaria and the inclusion of inadequate partner drugs with the artemisinins. Considering endemicity, patients with symptomatic malaria from highly endemic areas such as Africa generally have higher pretreatment parasite densities compared to patients from lower endemic areas such as Southeast Asia[3], and higher pretreatment parasite densities have been associated with a higher risk of recrudescence after treatment with AS [28]. Additionally, the increased risk of new infections in highly endemic areas may contribute greatly to treatment outcome. These factors suggest that in Africa, more so than other areas, it is critical that artemisinins be combined with other highly effective drugs. However, whether partner drug should be short- or long-acting remains unclear. Long-acting partner drugs offer an extended prophylactic effect; however, they may encourage the selection of drug resistance. Considering the efficacy of ACTs, initial studies in Africa combining AS with available drugs (CQ, SP, and AQ), were relatively disappointing in areas where the efficacy of the partner drugs was likely limited by resistance, as was seen in this study [18,26,27]. ACT regimens with more effective partner drugs (e.g., artemether-lumefantrine and/or dihydroartemisinin-piperaquine) will likely show improved efficacy [4].

Despite the limitations noted, ACT and other antimalarial combination therapies offer great hope for Africa. However, the ideal combination regimens remain uncertain [4]. As African countries rapidly move toward replacement of ineffective monotherapies with combinations regimens, it is essential that controlled trials be conducted to compare efficacy, ideally considering the importance of varied transmission intensity. Studies with extended follow-up and longitudinal designs will provide the most useful comparisons, as different therapies may impact very differently on long-term outcomes. Based on the results of this study and others, Uganda has recently opted to replace CQ + SP with artemether-lumefantrine, joining many other African countries in choosing an ACT as first-line therapy. However, cost and availability of ACTs remain major concerns, and it appears that the sudden increase in demand for artemisinins may exacerbate these problems, at least in the short-term [5]. The inexpensive and widely available combination AQ + SP may still be appropriate for the treatment of uncomplicated malaria in areas where resistance to the drugs is relatively uncommon, in situations where ACT may not be feasible (e.g., such as the treatment of unconfirmed cases of malaria outside of the formal health sector), and, in the short term, while adequate supplies of ACT are not yet available in Africa.

## Supporting Information

Protocol S1 CONSORT Checklist (58 KB DOC).

Protocol S2 Combination Therapies for Treatment of Complicated *falciparum* Malaria in Uganda: Evaluation of Efficacy, Safety, and Tolerability (1.0 MB DOC).

**Patient Summary**

**Background**

As resistance to current malarial regimens grows, it is increasingly important to find new combinations of drugs that will not only treat an ongoing infection, but will also prevent recurrence of the malaria (either a new infection or reappearance of the treated infection). One type of drug that is being assessed for preventing recurrence is based on artemisinin, which was originally derived from a plant, sweet wormwood. However, this drug is more expensive than older drugs

**What Did the Authors Do?**

They did four randomized clinical trials in Africa at the same time; two in areas where malaria occurs very frequently and two where it is less frequent. They compared a combination of artemisinin with two other combinations of older drugs and looked to see how well the treatments worked on the present infection, on preventing recurrences, and on whether there were any serious adverse events.

They found that the combination including artemisinin worked the best at treating current infections. However, patients given the artemisinin-based treatment were overall more likely to get new infections. Where malaria was very common, people treated with the artemisinin-based combination were more likely to get recurrent infections overall.

**What Do These Findings Mean?**

Although the artemisinin-based treatment worked very well on present infections, for recurrent infections it did not perform better than, and was considerably more expensive than, an older combination of drugs. Artemisinin-based treatment should not automatically be assumed the best treatment for uncomplicated malaria in Africa.

**Where Can I Get More Information?**

The World Health Organization has a Web site on Africa with links to malaria control:

http://www.afro.who.int/

The United Kingdom Department for International Development has information on malaria control in Africa:

http://www.lshtm.ac.uk/dfid/malaria/

## Abbreviations

ACT: artemisinin-based combination therapy
AQ: amodiaquine
AS: artesunate
CI: confidence interval
CQ: chloroquine
ETF: early treatment failure
LCF: late clinical failure
LPF: late parasitological failure
SP: sulfadoxine-pyrimethamine
