# Supplementary material for: Artemisinin versus Nonartemisinin Combination Therapy for Uncomplicated Malaria: Randomized Clinical Trials from Four Sites in Uganda
Source: PLoS Med. 2005 Jul 26;2(7):e190. doi: 10.1371/journal.pmed.0020190 (PMC1181876; doi:10.1371/journal.pmed.0020190)
Supplement: Protocol S2 — (1.0 MB DOC). [file pmed.0020190.sd002.doc]

**COMBINATION THERAPIES FOR TREATMENT OF UNCOMPLICATED FALCIPARUM MALARIA IN UGANDA:**

**EVALUATION OF EFFICACY, SAFETY, AND TOLERABILITY**

**Protocol version 1.1**

**Uganda Malaria Surveillance Project drug efficacy studies**

**DECEMBER 20, 2002**

TABLE OF CONTENTS.

1.0. Summary 4

2.0 INTRODUCTION 5

2.1 Background 5

2.2 Rationale 5

3.0 STUDY AIMS 5

4.0 STUDY DESIGN 5

4.1 Overall Design 5

4.2 Study outcome and measurement 6

4.2.1 Primary outcome 6

4.2.2 Secondary outcome 6

4.3. Randomization: 6

4.4. Treatment allocation and masking: 7

5.0 PARTICIPANT SELECTION AND ENROLLMENT 7

5.1. Recruitment and screening process: 7

5.2. Selection criteria: 8

5.3. Process for obtaining informed consent: 8

6.0 BASELINE EVALUATION AND TREATMENT ASSIGNMENT 9

6.1. Baseline evaluation and procedures: 9

6.2. Treatment group assignment 9

6.3. Study drug information 9

6.3.1. Study drug formulation and labeling 9

6.3.2. Study drug dosing schedule 10

7.0 FOLLOW-UP EVALUATION AND PROCEDURES 10

7.1. Follow-up schedule 10

7.2. Assessment for adverse events related to study medications: 11

7.2.1. Definitions of adverse events 11

7.2.2. Identification of adverse events 11

7.2.3. Reporting of adverse events 11

7.2.4. Management of severe or life-threatening adverse events 11

7.3. Management of clinical treatment failures: 11

7.4. Exclusion after enrollment and loss to follow-up 12

7.5. Pilot study: 12

8.0 STATISTICAL CONSIDERATIONS [12](#__RefHeading___Toc11143181)

8.1. Sample size calculations: [12](#__RefHeading___Toc11143182)

8.2. Analytical plan: 12

9.0 DATA COLLECTIONS AND MANAGEMENT [13](#__RefHeading___Toc11143184)

9.1. Data management: [13](#__RefHeading___Toc11143185)

9.2. Data quality assurance and monitoring: [13](#__RefHeading___Toc11143186)

9.3. Records: 13

9.4. Use of filter paper samples. 14

10.0 PROTECTION OF HUMAN SUBJECTS 14

10.1. Institutional Review Board (IRB) review and informed consent: [14](#__RefHeading___Toc11143189)

10.2. Evaluation of benefits and risks/discomforts: [14](#__RefHeading___Toc11143190)

10.3. Compensation: [14](#__RefHeading___Toc11143191)

10.4. Consent procedures: [14](#__RefHeading___Toc11143192)

10.5. Subject confidentiality: 15

11.0 REFERENCES 15

12.0. TIMETABLE 16

APPENDIX 1.STEPS THAT A PATIENT GOES THROUGH DURING THE STUDY 17

APPENDIX 2.CRITICAL STEPS. 18

APPENDIX 3.OUTCOME CLASSIFICATION 19-20

APPENDIX 4.WEIGHT-BASED ADMINISTRATION OF STUDY MEDICATIONS 21-22

APPENDIX 5.SYMPTOM GRADING. 23

APPENDIX 6.EXAMINATION GUIDELINES. 24-27

APPENDIX 7.CONSENT FORM TO PARTICIPATE IN A RESEARCH STUDY 28-39

APPENDIX 8. PATIENT SCREENING FORM 40

APPENDIX 9.ENROLLMENT FORM 41

APPENDIX 10. ADVERSE EVENT REPORTING GUIDELINES…………………………………...42-45

APPENDIX 11. SERIOUS ADVERSE EVENT REPORTS FORM…………………………………...46-47

APPENDIX 12. EXPECTED ADVERSE EVENTS FOR CHLOROQUINE……………………….…….48

APPENDIX 13. CRITERIA FOR SEVERE MALARIA/DANGER SIGNS . 49

APPENDIX 14. UMSP ADVERSE EVENT FOLLOW-UP GUIDELINES. 50

APPENDIX 15. UMSP CLINICALRECORD FORMS………………………………………………...51-58

APPENDIX 16. UMSP ADVERSE EVENT RECORD FORM..…………………………………………..59

**1.0. Summary**

| **Title** | Combination therapies for treatment of uncomplicated malaria in Uganda: evaluation of efficacy, safety, and tolerability |
| --- | --- |
| **Description** | Randomised, single-blinded trials comparing combination antimalarial regimens for treatment of uncomplicated falciparum malaria with 28-day follow-up. |
| **Participants** | Ugandans age > 6 months. |
| **Clinical Sites** | The studies will be conducted at the seven Uganda Malaria Surveillance Project (UMSP) sentinel sites. The Ugandan Ministry of Health (MoH) originally established these sites in 1998 in collaboration with the East African Network for Monitoring Antimalarial Treatment (EANMAT). |
| **Selection Criteria** | 1. Age > 6 months. 2. Fever (> 37.5ºC axillary) or history of fever in the previous 24 hours. 3. Absence of any history of serious side effects to study medications, including allergy to sulfa drugs 4. No evidence of severe malaria or danger signs 5. No evidence of a concomitant febrile illness 6. *P. falciparum* mono-infection 7. Parasite density > 2000/ul and < 200,000/ul 8. Agreement to return for all scheduled follow-up visits 9. Provision of informed consent 10. No history of anti-folate or amodiaquine use in past 7 days 11. Absence of pregnancy |
| **Follow-up** | Subjects will be asked to return on Days 1, 2, 3, 7, 14, 21, 28, and any unscheduled day that they feel ill for follow-up assessment. |
| **Study objectives** | To assess the efficacy, safety and tolerability of alternative antimalarial therapies for treatment of uncomplicated falciparum malaria as they compare to chloroquine/sulfadoxine-pyrimethamine (CQ/SP) treatment. |

### 2.0 INTRODUCTION

### 2.1 Background

Malaria remains one of the most serious global health problems and a leading cause of morbidity and mortality in Uganda. Appropriate case management, focusing on prompt treatment with effective antimalarial drugs, is the foundation of malaria control throughout sub-Saharan Africa. For decades, chloroquine (CQ) was the mainstay of antimalarial therapy, but the emergence of *P. falciparum* resistance to CQ has challenged control efforts.1 The spread of CQ resistance has been temporally associated with increased malaria-related morbidity and mortality in Africa, highlighting the urgent need to change antimalarial treatment policy in the face of rising CQ resistance.2 Unfortunately, the optimal alternative to CQ is not clear and available drugs are limited by cost, efficacy, and safety concerns. In 2000, the Ugandan MoH re-evaluated the national antimalarial drug policy in response to expanding CQ resistance. The combination of CQ plus sulfadoxine-pyrimethamine (SP) was chosen to provisionally replace CQ as first-line treatment for uncomplicated malaria. However, at the time that this decision was made, the efficacy and safety of the CQ/SP combination had not been evaluated in Uganda and it is anticipated that pre-existing resistance to both agents will limit the effective lifespan of this combination. To facilitate rational drug policy decision-making in the future, the investigation of alternative antimalarial regimens must continue. Regimens currently under consideration in Uganda for treatment of uncomplicated malaria include amodiaquine (AQ) plus SP, artesunate (AS) plus SP, and AS + AQ. Other possible therapies include artemether-lumefantrine (Co-Artem), and chlorproguanil/dapsone (Lapdap) combinations, when this agent is available.

Malaria control in East Africa is approaching a crisis. Use of CQ to treat malaria has failed on a public health level. SP resistance is already increasing and is likely to spread rapidly. Combination treatment with currently available monotherapies appears to be the most promising hope for treatment of malaria in the region. The lack of therapeutic options and need to confront rising CQ resistance and resultant increase in malaria-associated morbidity and mortality is forcing East African countries, including Uganda, to consider the introduction of combination antimalarial therapies on a large scale. In the absence of adequate data on the tolerability and efficacy of these regimens, the need for additional data on combination antimalarial therapy cannot be overstated.

### 2.2 Rationale

We are proposing to conduct drug efficacy studies to evaluate the efficacy and safety of combination antimalarial regimens for treatment of uncomplicated falciparum malaria in Ugandans. The choice of combination regimens to be tested in these studies will be driven by alternative antimalarial regimens prioritised by the Ugandan MoH and EANMAT. Generally, CQ + SP will serve as the standard treatment and will be compared to one or more alternative regimens including AQ/SP and AQ/AS. The data that is collected in these studies will be made directly available to the Ugandan MoH to assist in drug policy decision-making.

### 3.0 STUDY AIMS

To assess the efficacy, safety, and tolerability of alternative combination antimalarial therapies as they compare to the standard CQ/SP treatment.

### 4.0 STUDY DESIGN

### 4.1 Overall Design

These studies shall be randomised, single-blinded trials designed according to World Health Organization (WHO) guidelines for assessment of therapeutic efficacy of antimalarial agents in areas of intense transmission^3^ with slight modifications. The target population is residents of the catchment areas of the sentinel health centres. The available population is residents aged 6 months and above who have symptoms suggestive of malaria and a positive screening thick blood smear and who are referred to the clinics for screening. Subjects who meet the inclusion criteria and are enrolled in the trial will be randomised to treatment with one of the combination regimens and will be followed for 28 days. Repeat evaluations will be performed on days 1, 2, 3, 7, 14, 21, and 28 and will include assessment for the occurrence of any possible adverse event. Treatment efficacy outcomes will be assessed using modifications of WHO clinical and parasitological classification criteria (Appendix 3).

### Study outcome and measurement

### 4.2.1 Primary outcome

Response to treatment will be classified according to WHO criteria (Appendix 3), using clinical (1996: ACR, ETF, LTF) and parasitological (S, RI/RII/RIII) classification systems, with slight modifications.^1^ In addition, patient outcomes will be classified using the newly proposed WHO classification system (2002: ACPR, ETF, LCF, LPF), for purposes of data reporting.^2^ The efficacy of the combination regimens will be assessed by comparing the proportion of patients with clinical treatment failure at 28 days. Clinical outcome will be dichotomised into success (ACR) and failure (ETF or LTF). Pair wise comparisons between regimens will be made on an intention-to-treat basis.

### 4.2.2 Secondary outcome

The efficacy of the combination regimens will also be assessed by comparing the treatment groups as follows:

1. Proportion of patients with clinical failure (ETF/LTF) at 14 days of follow-up (Appendix 3)
2. Proportion of patients with parasitological resistance (RI/RII/RIII) at 14 and 28 days of follow-up (Appendix 3) to supplement results of clinical outcome and to facilitate comparison with historical data.
3. Proportion of patients with treatment failure according to new WHO guidelines (ETF/LCF/LPF) at 14 and 28 days of follow-up (Appendix 3).
4. Rate of fever clearance: presence or absence of objective fever (axillary temperature > 37.5°C) or patient report of fever on days 1, 2, 3
5. Rate of parasite clearance: proportion of positive vs. negative thick blood smears on day 2 and day 3
6. Change in mean haemoglobin from day 0 to 28
7. Proportion gametocytemic: presence vs. absence of gametocytes on any follow-up thick blood smear; proportion gametocytemic on days 2, 3, 7, 14, 21, and 28.

The safety and tolerability of the combination regimens will be further compared as follows:

1. Incidence of serious adverse events (AEs): proportion of patients experiencing any serious AE in each treatment group during the 28-day follow-up period.
2. Incidence of AEs: proportion of patients experiencing any mild/moderate/severe/life-threatening AEs during the 28-day follow-up period
3. Incidence of AEs by specific sign or symptom during the 28-day follow-up period

### 4.3. Randomization:

An age-stratified approach to treatment group assignment using two age groups (6-59 months and 5 years and above) will be used. Randomisation lists shall be generated in the Kampala office by a member of the project who will not be directly involved in the conduct of the study. Two separate allocation schedules (A and B) shall be generated. A sealed copy of the original randomisation lists and documentation of the procedure used to generate the lists will be stored in the project administrative offices in Kampala. Prior to the onset of the study, sealed copies of the randomization lists will be distributed to the study nurse responsible for treatment allocation.

### 4.4. Treatment allocation and masking:

On the day of enrolment, study participants will undergo baseline evaluations and be assigned a study number by the clinicians. Patients will then be referred to the study nurse who will be responsible for the treatment group assignment and allocation of the study medications. The study nurse will note the patient’s age and select the next available treatment number and corresponding assigned treatment. The study nurse will record directly onto the randomization list the date and time of treatment assignment and study number. Once a month, a copy of the updated randomization list will be placed by the study nurse into sealed envelopes and delivered to the administrative office in Kampala where they will be stored securely. The original randomization list will be crosschecked to assure adherence to the randomisation code. Initial studies will involve one of the two following treatment comparison designs:

1. Two-arm study – CQ/SP vs. AQ/SP
2. Three-arm study - CQ/SP vs. AQ/SP vs. AQ/AS

Sentinel sites with previously identified low levels of drug resistance and/or lower-level capacity for the conduct of complex studies will be chosen for the two-arm study. Sentinel sites with previously identified higher levels of drug resistance and greater capacity will be chosen for the three-arm study.

The nurse shall administer study medications according to weight-based guidelines (Appendix 4). Study medications will not be identical in appearance or taste, however other procedures will be followed in an attempt to mask the patients to their treatment. Patients will not be informed of their treatment regimen and identical dosing regimens will be used in all the treatment arms. In studies where AS/AQ shall be compared with CQ/SP and AQ/SP, patients assigned to CQ/SP and AQ/SP shall be given a placebo (lactose tablets) on day 1 and day 2 in the same amount as AS. Administration of study medications will be directly observed. Any patient who vomits the medication within 30 minutes of administration of the drugs will be retreated with a second dose. If the patient vomits repeatedly on Day 0, they shall be excluded. If the patient vomits repeatedly on Day 1 or 2 and is unable to retain the dose of the study medication, the patient will be classified as a treatment failure and referred for appropriate care with parental antimalarial medication.

All members of the study group who will be involved in assessment of patient outcomes following treatment for uncomplicated malaria including the study physicians (responsible for clinical assessment and measurement of temperature) and laboratory technicians (responsible for reading thick blood smears and determining parasite density) will be blinded to the treatment group assignments.

### 5.0 PARTICIPANT SELECTION AND ENROLLMENT.

### 5.1. Recruitment and screening process:

Study subjects will be recruited from the OPD (outpatient department) of sentinel health centres. Patients who present with symptoms suggestive of malaria (fever or history of recent fever) will be referred to the outpatient laboratory/study laboratory for a screening thick blood smear (using standard Giemsa staining). Thick blood slides will be read and counted by the outpatient/study laboratory technicians. The parasite density of positive screening thick blood smears will be estimated by the outpatient laboratory technicians by counting the number of asexual parasites per 200 leukocytes, assuming a leukocyte count of 8,000/μl. All patients who have a positive screening thick smear with a parasite density of > 2000/ul (>13 parasites per 200 leucocytes) and < 200,000/ul will be referred for further evaluation. After referral to the study clinic, the screening interview will take place. If the patient satisfies the selection criteria, they will be enrolled in the study. All patients who do not satisfy the selection criteria and are excluded from study enrolment will be referred back to the outpatient department for appropriate care.

### 5.2. Selection criteria:

Patients shall be assessed for the inclusion criteria at the time they are referred to the study clinic with a screening thick blood smear parasitaemia of > 2000/ul and < 200,000/ul. If the following criteria are met, patients will be enrolled in the study, assigned a study number, have blood removed by finger-prick for further laboratory tests (Giemsa-stained thick and thin blood smears, Hb measurement and blood collected on filter paper) and will be treated promptly according to their assigned antimalarial study regimen.

1. Age 6 months and above.
2. Fever (> 37.5ºC axillary) or history of fever in the previous 24 hours.
3. Absence of any history of serious side effects to study medications, including allergy to sulphonamide drugs
4. No evidence of a concomitant febrile illness which could interfere with the assessment of clinical treatment outcome
5. Provision of informed consent and ability to participate in 28-day follow-up (patient has easy access to health unit).
6. No history of treatment with an antifolate (sulfadoxine-pyrimethamine [single dose], metakelfin [single dose], TMP/Sulfa [> 3 days]) or amodiaquine (> 2 doses) during the last week (taking final dose within the 7 days prior to study enrolment).
7. Absence of pregnancy (menstruating women who have not missed their last menstrual period).
8. No danger signs or evidence of severe malaria defined as:

- Unarousable coma (if after convulsion, > 30 min)
- Repeated convulsions (> 2 within 24 h)
- Recent convulsions (1-2 within 24 h)
- Altered consciousness (confusion, delirium, psychosis, coma)
- Lethargy
- Unable to drink or breast feed
- Vomiting everything
- Unable to stand/sit due to weakness
- Severe anemia (Hb < 5.0 g/dL)
- Respiratory distress (laboured breathing at rest)
- Jaundice (yellow coloring of eyes)

Prior to the Day 1 return visit, the study laboratory technicians shall assess the results of the Giemsa-stained thick and thin blood smears. These shall not be available until after the patients have been treated and discharged from the clinic. Additional patients will be excluded from the study on Day 1 if they fail to meet the following additional inclusion criteria.

1. *P. falciparum* mono-infection
2. Parasite density > 2000/ul and < 200,000/ul.

Any patient who is excluded on Day 1 will be treated and followed-up in a clinically appropriate manner, but will not be followed for the 28-day period. The study number and treatment number of a patient excluded on Day 1 will not be re-assigned.

5.3. Process for obtaining informed consent:

Study clinicians shall conduct the screening interview and seek formal consent in the clinic. Once it has been determined that the subject meets the study entry criteria during the screening interview, all study details will be carefully discussed with the subject or their parents/guardian. All interviews shall be conducted in the native language of the patients and parents/guardians of children. Consent forms will be available in both English and the local language. Following the informed consent discussion, the patient or parents /guardians of children shall be asked to sign a written consent form. If they are unable to read and write, a fingerprint will be used in substitute for a signature and a witness will be asked to sign the form to document that the informed consent process was adequately conducted.

### 6.0 BASELINE EVALUATION AND TREATMENT ASSIGNMENT

### 6.1. Baseline evaluation and procedures:

On Day 0, patients enrolled and assigned study numbers will undergo a complete history and physical examination (Appendix 5 and 6). On day 0, a finger-prick blood sample will be obtained for repeat thick blood smear, thin blood smear, haemoglobin measurement and to save on filter paper for future molecular testing. Because only rapid Giemsa-stained thick smears are typically assessed in the outpatient laboratory, a thick and thin blood smear obtained at enrolment will be stained with 2% Giemsa and read by experienced laboratory technicians who are not involved in direct patient care. Parasite densities will be calculated by counting the number of asexual parasites per 200 leukocytes (or per 500, if the count is <10 parasites/200 leukocytes), assuming a leukocyte count of 8,000/μl. A blood smear will be considered negative when the examination of 100 high power fields does not reveal asexual parasites. Gametocytemia will also be determined from thick smears. Thin smears will be used for parasite species identification.

### 6.2. Treatment group assignment:

At the time of enrolment, patients will be randomly assigned to one of the treatment groups. Randomisation shall be according to a pre-determined randomisation list stratified by age. Treatment allocation and administration of medications will be done by the study nurse who will not be involved in outcome classification and will remain the only member of the study team not blinded to the treatment groups.

Administration of study medications will be performed by the study nurse and will be directly observed (Appendix 4). Any patient who vomits the medication within 30 minutes of administration of the drugs will be retreated with a second dose. If the patient vomits repeatedly on Day 0, and is unable to retain the dose of the study medication, the patient will be excluded from the study. If this occurs on Days 1 or 2, the patient will be classified as a treatment failure, referred for appropriate care and treated with parenteral antimalarial medication.

Patients will receive acetaminophen 10 mg/kg with instructions to take it every 8 hours until the resolution of fever. Any child with a hemoglobin of < 10.0 gm/dl will be treated with iron sulfate for 2 weeks and mebendazole (only those aged 1 - 5 years).

### 6.3. Study drug information:

### 6.3.1 Study drug formulation and labeling:

| **Drug** | **Trade name**  **Manufacturer** | **Class** |
| --- | --- | --- |
| Amodiaquine (200mg) | Camoquin  Parke-Davis | 4-aminoquinoline |
| Artesunate (50mg) | Arsumax  Sanofi/Winthrop | Artemisinin derivative |
| Chloroquine (250 mg) | Cosmoquin  Cosmos, Kenya | 4-aminoquinoline |
| Sulfadoxine-pyrimethamine  (500mg/25mg) | Fansidar  Roche | Antifolate combination |

### 6.3.2 Study drug dosing schedule:

| **Treatment Groups** | **Medications** | **Day 0** | **Day 1** | **Day 2** |
| --- | --- | --- | --- | --- |
| CQ/SP | CQ | 10 mg/kg | 10 mg/kg | 5 mg/kg |
|  | SP | 25 mg/kg sulfa component | Placebo if 3-arm  study | Placebo if 3-arm  study |
| AQ/SP | AQ | 10 mg/kg | 10 mg/kg | 5 mg/kg |
|  | SP | 25 mg/kg sulfa component | Placebo if 3-arm  study | Placebo if 3-arm  study |
| AS/AQ | AS | 4 mg/kg | 4 mg/kg | 4 mg/kg |
|  | AQ | 10 mg/kg | 10 mg/kg | 5 mg/kg |

### FOLLOW-UP EVALUATION AND PROCEDURES

7.1. Follow-up schedule:

Patients will go home on Day 0 and will be asked to return to the clinic for follow-up on Days 1, 2, 3, 7, 14, 21, 28, and any unscheduled day that they feel ill. All patients will be reimbursed the cost of their transport to and from the clinic. Details about the location of the patient’s residence will be obtained and if a subject does not return for a scheduled clinic follow-up appointment, the study personnel shall visit them at home. Patients who return on Day 1 and fail to fulfil the criteria of *P. falciparum* mono-infection with a parasite density of > 2000 parasites/ul and < 200,000 parasites/ul will be excluded from further study and referred to OPD for management. At each repeat visit, temperature will be measured and a focused physical examination will be performed. A finger prick blood sample will be obtained on Days 2, 3, 7,14, 21 and 28 (and any extra day) to repeat thick blood smears and to save on filter paper. Haemoglobin will be re-evaluated either on day 28 or at the time of clinical treatment failure. All thick smears will be assessed for parasite density and presence of gametocytes. Any patient who is suspected of clinical treatment failure after Day 3 will have a repeat thick and thin smear obtained.

Follow-up Schedule

|  | **Day 0** | **Day 1** | **Day 2** | **Day 3** | **Day 7** | **Day 14** | **Day 21** | **Day 28** | **Extra Day** | **When necessary** |
| --- | --- | --- | --- | --- | --- | --- | --- | --- | --- | --- |
| Study medications | X | X | X |  |  |  |  |  |  |  |
| History | X | X | X | X | X | X | X | X | X |  |
| Temperature measurement | X | X | X | X | X | X | X | X | X |  |
| Physical exam | X | X | X | X | X | X | X | X | X |  |
| Neurologic exam (3-arm study only) | X |  |  |  | X | X | X | X | * |  |
| Thick blood smear | X |  | X | X | X | X | X | X | X |  |
| Thin blood smear | X |  |  |  |  |  |  |  |  | CTF after day 3 |
| Filter paper sample | X |  |  | X | X | X | X | X | * |  |
| Haemoglobin | X |  |  |  |  |  |  | X | * | If CTF, on day of failure |
| Assessment for adverse drug event | X | X | X | X | X | X | X | X | X |  |

X = perform this task

* As indicated

### 7.2. Assessment for adverse events related to study medications (Appendix 10):

### 7.2.1 Definitions of adverse events:

A description of expected adverse events for each of the study medications shall be obtained from package inserts of the study medications. The following definitions will be used according to NIAID guidelines:

- Adverse event: any untoward medical occurrence, including dosing errors, that may arise during administration of study agent, and which may or may not have a causal relationship with the study agent.
- Unexpected adverse event: any adverse experience that has not been previously observed (i.e. Included in the labeling), whether or not the event is anticipated because of the pharmacologic properties of the study agent
- Serious adverse event: any adverse experience that results in any of the following outcomes: death; life threatening experience; inpatient hospitalization; persistent or significant disability or incapacity; or specific medical or surgical intervention to prevent one of the other serious outcomes listed in the definition.

### 7.2.2. Identification of adverse events:

At each follow-up visit (days 1, 2, 3, 7, 14, 21, 28, and any unscheduled day), study clinicians will assess patients according to a standardized clinical record form (Appendix 14). All signs and symptoms present will be graded as mild, moderate, severe, or life threatening according to examination guidelines (Appendix 6). An AE will be defined as a sign or symptom of untoward medical occurrence arising or increasing in severity compared to the previous day’s record.

**7.2.3 Reporting of adverse events:**

For each possible adverse event identified and graded as moderate, severe or life threatening, an adverse event report form will be completed (Appendix 15). An adverse event report form will not be completed for events classified as mild as these symptoms are common and difficult to distinguish from signs and symptoms due to malaria (however, mild AEs will still be included in the analysis). The following information will be recorded for all adverse experiences that are reported:

1. Description of event
2. Date of event onset
3. Date event reported
4. Maximum severity of the event
5. Maximum suspected relationship of the event to study medication
6. Is the event serious?
7. Initials of the person reporting the event
8. Was the event episodic or intermittent in nature?
9. Outcome
10. Date event resolved

Serious adverse events occurring in the trial shall be reported to the core facility by telecommunication within 24 hours. All information submitted by the surveillance sites will be collected at the Kampala core facility and reported to the Ugandan National Drug Authority and MoH.

### 7.3. Management of clinical treatment failures:

Patients who are classified as clinical failures (early or late treatment failure, Appendix 3) will be treated with quinine 10 mg/kg orally three times a day for 7 days. Any patient who develops evidence of severe malaria or danger signs during malaria follow-up, will be referred for appropriate treatment with parenteral quinine at the local facility or hospital. Any patient who responds clinically to treatment, but has a positive thick blood smear on day 28 (asymptomatic parasitaemia) will be treated with standard doses of oral quinine. Hemoglobin will be repeated on the day the patient is classified as a clinical treatment failure.

- 1. **Exclusion after enrolment and loss to follow-up:**

Patients will be excluded from the study after enrolment if any of the following occurs:

- 1. Use of antimalarial drugs outside of the study protocol.
  2. Development of concomitant febrile illness that may interfere with outcome classification.
  3. Withdrawal of informed consent.

Patients who fail to attend a follow-up visit and are unable to be located within 24 hours on Days 1-3 or within 48 hours on Days 4-28 will be considered lost to follow-up.

### 7.5. Pilot study:

A pilot study is planned prior to the onset of the actual study at each site. Approximately 10 pilot subjects shall be enrolled. They shall undergo all study procedures and will be followed for the full 28-day study period. A study group meeting will be held one week after beginning the pilot study to review all study procedures and assess for potential difficulties. We anticipate that actual study enrolment will begin 2 weeks after enrolling the pilot subjects.

### 8.0 STATISTICAL CONSIDERATIONS

### 8.1. Sample size calculations:

The sample size calculations will be made for each study based on the estimated efficacy of the combination regimens to be studied in the given region. For the purposes of sample size calculation, the risk of clinical failure at 28 days will be the main outcome. If the null hypothesis states that there is no difference in clinical outcome between the treatment groups, we plan to test the alternative hypothesis that treatment with an alternative combination regimen (AQ/SP or AS/AQ) will decrease the likelihood of clinical failure at 28 days of follow-up. Calculations will be made using an α = 0.05 and a statistical power of 80% based on a two-sided test. Allowance will be made for a possible 10% loss to follow-up to ensure that an adequate number of patients complete the study. Based on this, the sample size for the various drug combinations is summarized below:

Sample size for the different drug combinations

| **Presumed level of resistance.** | Resistance to CQ/SP | Resistance to AQ/SP or AS/AQ | Number per group | Number per group adjusted for loss to follow up. |
| --- | --- | --- | --- | --- |
| High resistance community (urban areas) | 15% | 5% | 160 | 180 |
| Low resistance community (rural areas) | 10% | 2% | 162 | 180 |

### 8.2. Analytical plan:

Data analysis will be primarily performed by the project statistician using EPI INFO, SPSS ACCESS and STATA statistical software packages, with additional assistance from members of the UCSF Epi-Center in San Francisco and the University of California, Berkeley, Department of Epidemiology. Primary analyses will be conducted based on intention-to-treat and the primary analysis population will include all randomised subjects fulfilling selection criteria.

Descriptive statistics will be used to summarise baseline characteristics of study patients. Parasite density values will be normalised using logarithmic transformation. All dichotomous variables will be compared between treatment groups using chi-square tests or Fisher’s exact tests. Continuous variables will be compared using t-tests. A p-value of < 0.05 will be considered significant.

Because age is a strong effect modifier in malaria, an age-stratified subgroup analysis for efficacy outcomes is planned. Study subjects will be stratified by age (< 5 years and > 5 years) for analysis of treatment outcome including clinical outcome at 14 and 28 days, and parasitological outcome at 14 and 28 days. Results for the two age groups will be presented independently. After calculating an estimate of association for each of the two age groups, a single adjusted measure of risk difference will be calculated. Other factors that may impact on antimalarial treatment outcome include temperature^4^ and mean parasite density at presentation (Dorsey, unpublished data). Because a randomised trial is planned, we anticipate that known and unknown confounding factors will be equally balanced between the treatment groups. If by chance, there is an imbalance between the treatment groups for one or more potential confounding variables, adjustment will be made using multiple logistic regression.

### 9.0 DATA COLLECTION AND MANAGEMENT

### 9.1. Data management:

All clinical data will be recorded onto standardised case record forms by study clinicians. Laboratory data will be recorded in a laboratory record book by the study laboratory technicians and then transferred to the case record forms by the study co-ordinator, who will review the case record forms frequently for completeness and accuracy. Data will be transferred from the case record forms into a computerised database (EPI INFO 6.04 or Access) by data entry personnel and will be double entered to verify accuracy of entry. Two back-up files of the database will be stored on zip discs after each data entry session. For quality control, check programs will be written into the database to limit the entry of incorrect data and ensure entry of data into required fields.

### 9.2. Data quality assurance and monitoring:

All members of the study team will be educated in the study protocol prior to the onset of the trial. Knowledge of the study protocol and procedures will be assessed and documented with a post-training questionnaire. The study clinicians will complete case record forms at each patient visit. These forms will be reviewed by the study co-ordinator for completeness and accuracy. For quality control of thick blood smear slide readings, an expert microscopists who will be blinded to the patient’s treatment group will review a random sample of approximately 10% of slides. A set of standard slides with known parasite densities will be sent through the laboratory periodically to ensure continued accuracy. Study group meetings will be conducted by the co-ordinator once a week to assess progress of the study, address any difficulties, and provide performance feedback to the members of the study group. In addition members from the core facility will make regular visits to active study sites as needed.

### 9.3. Records:

Case record forms will be provided for each subject. Participants will be identified by their names and study identification number on the case record form. Patient names will not be entered into the computerised database. All patient record forms will be kept in individual files in a secure filing cabinet in the study clinic. All corrections will be made on case record forms by striking through the incorrect entry with a single line and entering the correct information adjacent to it. The correction will be initialled and dated by the investigator. Any requested information that is not obtained as specified in the protocol will have an explanation noted on the case record form as to why the required information was not obtained. Additional records will be kept in the clinical and laboratory record books that will be stored in the central study laboratory in Makerere University Medical School. The investigators will allow all requested monitoring visits, audits or reviews.

# **9.4. Use of filter paper samples**: Blood samples will be collected from the patients enrolled in the clinical study on days 0, 3, 7, 14, 21, 28 and on any unscheduled day when the patient presents with clinical deterioration or recurrent fever. Blood will be placed onto filter paper in approximately 25 ul aliquots per blood spot (4 blood spots per sample). The samples will be labeled, air-dried and stored in small, sealed sample bags at ambient temperature. Parasite DNA will subsequently be removed from the filter paper and prepared for molecular analysis using a chelex extraction method. Two types of molecular studies of the parasites collected during this clinical study are planned: 1) Genotyping of parasites collected at baseline (day 0) and during follow-up to distinguish between true recrudescence and reinfection with new parasites. Analysis will be done using a nested PCR technique targeting polymorphisms in MSP-1, MSP-2, and GLURP parasite genes to assess for molecular markers of drug resistance. 2) Analysis of polymorphisms in parasite DHFR/DHPS genes will be done using a nested PCR technique to assess for key mutations associated with SP resistance. 3) Analysis for polymorphisms in the pfcrt and pfmdr-1 genes will be done to assess for mutations associated with 4-aminoquinoline (CQ and AQ) resistance. This laboratory work will be conducted in the UCSF laboratory directed by Dr. Phil Rosenthal and in the Makerere University laboratory directed by Dr. Fred Kironde.

### 10.0 PROTECTION OF HUMAN SUBJECTS

### 10.1. Institutional Review Board (IRB) review and informed consent:

This protocol and the informed consent documents, including any additional educational or recruitment material will be reviewed and approved by the Uganda National Council of Science and Technology (UNCST), the UCSF Committee for Human Research, and the UC Berkeley IRB before beginning the trial. Any amendments or modifications to this material will also be reviewed and approved by the IRB prior to implementation.

### 10.2. Evaluation of benefits and risks/discomforts:

Benefits that patients are likely to receive from participation in the study include enhanced medical attention, prompt diagnosis, free treatment, and close follow-up. Potential risks include drug side effects (described with chemoprophylactic use of AQ and SP) including hepatotoxicity, agranulocytosis, severe mucocutaneous reactions, and death. Although neurotoxicity associated with AS has been reported in animals, there is currently no evidence of such toxicity in humans.^5-8^

### 10.3. Compensation:

The patients/patient’s families will receive reimbursement for transportation costs to and from the clinic. In addition, all clinic visits, antimalarial medication, and the evaluation and treatment for some routine medical problems encountered during follow-up will be provided free of charge. Medical care that the patient receives which is unrelated to malaria will remain the primary responsibility of the patient, parent or guardian, although routine medical problems will generally be managed by the study at no cost to the patient.

### 10.4. Consent procedures:

All screening interviews will be conducted in the native language of the patients by the study personnel (with a translator if necessary). Consent forms will be provided to the patients/ parents or (guardians) for their review. The study clinicians at the clinic will seek formal consent. The patients / parents or (guardians) will be asked to sign consent to participate in a research study. The informed consent will describe the purpose of the study, the procedures to be followed, and the risks and benefits of participation). If the patient, parent or (guardian) is unable to read or write, their fingerprint will be used in substitute for a signature, and a signature from a witness to the informed consent discussion will be obtained. The patients / parents or (guardians) will be informed that the participation in the study is completely voluntary and that they may withdraw from the study at any time.

### 10.5. Subject confidentiality:

The patients/parents (guardians) will be informed that participation in the research study may involve a loss of privacy, however, only study personnel will have access to the medical information collected about the patient. All records will be kept as confidential as possible and patients will be identified primarily by their study number. No individual identities will be used in any reports or publications resulting from the study.

1. **REFERENCES**

1. Campbell CC. Challenges facing antimalarial therapy in Africa. *Journal of Infectious Diseases* 1991;**16:**1207-1211.

2. Trape JF. The public health impact of chloroquine resistance in Africa. *Am J Trop Med Hyg* 2001;**64**(1,2 Suppl)**:**12-17.

3.World Health Organization. Assessment of therapeutic efficacy of antimalarial drugs for uncomplicated falciparum malaria in areas with intense transmission. Geneva, 1996.

4. Dorsey G, Kamya MR, Ndeezi G, et al. Predictors of chloroquine treatment failure in

children and adults with falciparum malaria in Kampala, Uganda. *Am J Trop Med*

*Hyg* 2000;**62**(6)**:**686-92.

5. Dayan A. Neurotoxicity and artemisinin compounds: do the observations in animals justify the limitation of clinical use? *Med Trop* 1998; **58**(3 Suppl)**:** 32-37.

6. Nontprasert A, Nosten-Bertrand M, Pukrittayakamee S, Vanijanonta S, Angus BJ, White N. Assessment of the neurotoxicity of parenteral artemisinin derivatives in mice. *Am J Trop Med Hyg* 1998; **59**(4)**:** 519-22.

7. Nontprasert A, Pukrittayakamee S, Nosten-Bertrand M, Vanijanonta S, White N. Studies of the neurotoxicity of oral artemisinin derivatives in mice. *Am J Trop Med Hyg* 2000; **62**(3)**:** 409-12.

8. Genovese R, Newman D, Brewer T. Behavioral and neural toxicity of the artemisinin antimalarial, arteether, but not artesunate and artelinate, in rats. *Pharmacol Biochem Behav* 2000; **67**(1)**:** 37-44.

### 12.0 TIMETABLE

# Table 1. Timetable for Study Activities

|  | **2002** | **2003** | **2004** |
| --- | --- | --- | --- |
|  | **JFMAMJJASOND** | **JFMAMJJASOND** | **JFMAMJJASOND** |
| **Finalization/approval of study protocol** | XXXXX |  |  |
| Database design | XXX |  |  |
| **Personnel training** | XXX |  |  |
| **Pilot study** | XXX |  |  |
| **Recruitment** | XXXXXX | XXXXXXXXXXXX | XXXXXXXXX |
| **Follow-up** | XXXXXX | XXXXXXXXXXXX | XXXXXXXXX |
| **Data entry/analysis** | XXXXXX | XXXXXXXXXXXX | XXXXXXXXX |
| **Manuscript preparation** |  | XXXXXXXXXXXX | XXXXXXXXXXXX |

# **Target population**

Patients > 6 months with uncomplicated malaria

Living in Sentinel site health center catchment area

**Excluded**

Refer back to OPD for appropriate treatment

Re-assign study number to next study subject

### Appendix 1: Participant Selection and Enrollment

# **Available population**

Patients > 6 months presenting to OPD with symptoms positive screening thick blood smear **(2000/ul – 200,000/ul)**

## Yes

## Yes

## Yes

## No

## No

## No

## No

Day 1

Meet final inclusion criteria?

1. *P. falciparum* monoinfection
2. Parasite density > 2000/ul and < 200,000/ul (Giemsa-stained thick smear)

Excluded on Day 1

Treat and follow-up appropriately

Do **not** re-assign study or treatment number

**Continue with scheduled follow-up**

### No

### Yes

**Screening and recruitment**

Complete screening form

**Informed consent process**

Study number assigned

**Initial enrollment**

Complete Enrollment and CRFs

Perform baseline clinical evaluations

**Randomization and Treatment Allocation**

Treatment number and code selected on basis of subject’s age

Study medication administered by nurse

**Completion of Day 0 visit**

Baseline laboratory evaluations performed

Scheduled for follow-up on Days 1, 2, 3, 7, 14, 21, 28

## Yes

**Persistent vomiting on Day 0**

Re-assign Treatment Number

### APPENDIX 2. CRITICAL STEPS.

Day 1.

Evaluate patient and complete Case Record Form. Record temperature and fever history

Administer study medications (DOT)

Thick smear if severe disease/danger signs

Day 2.

Evaluate patient and complete Case Record Form. Record temperature and fever history, administer study medications (DOT)

Thick smear if severe disease/danger signs or temperature > 37.5 ^0^ C Collect thick blood smear and filter paper sample

Day 3.

Evaluate patient and complete Case Record Form. Record temperature and fever history Collect thick blood smear and filter paper sample

Day 7, 14, 21. Evaluate patient and complete Case Record Form. Record temperature and fever history, collect thick blood smear and filter paper sample

Any Unscheduled Day.

Evaluate patient and complete Case Record Form. Record temperature and fever history. Collect thick blood smear with filter paper sample.

**Parasitaemia > day 0 and**

**Temperature > 37.5^0^ C.**

**ETF**

Treat with quinine.

**Severe disease or danger signs**

Urgent reading of thick blood smear

**Temperature**

**> 37.5^0^C** **with parasitaemia**

**Parasite count >25% Day 0 count.**

**ETF**

Treat with quinine

Day 28.

Evaluate patient and complete Case Record Form. Record temperature and fever history, collect thick blood smear and filter paper sample.

If there is

**Severe disease or danger signs**

**Temp > 37.5°C**

**3. History of fever in**  **the past 24 hours.**

Collect thin blood smear.

Urgent reading of thick blood and thin blood smear

**Negative**

Assess for DDx

Continue study Rx

Refer for care?

**Negative**

Assess for DDx

Cont. study Rx

Refer for care?

**Positive blood smear**

**ETF**

Rx with quinine

Refer for care?

**Positive blood smear**

**LTF**

Rx with quinine

Refer for care?

**ACR**

No parasitaemia on day 28 irrespective of fever.

Temperature < 37.5^0^C and no history of fever in the last 24 hours irrespective of parasitaemia.

# Day 2 > Day 0

## ETF

Rx with quinine

Refer for care?

# Day 2 < Day 0

Assess for DDx

Cont. study Rx

Refer for care?

# Yes

## ETF

Rx with quinine

'

### APPENDIX 3. OUTCOME CLASSIFICATION

### Clinical Outcome Classification System

| ACR (Adequate Clinical Response)   - Absence of parasitemia on Day 28, without previously meeting criteria for ETF or LTF - Temperature < 37.5°C (Axillary) and no history of fever in last 24 hours on day 28 irrespective of the presence of parasitemia, without previously meeting any of the criteria of ETF or LTF |
| --- |
| ETF (Early Treatment Failure): Days 0, 1, 2, and 3  - Development of danger signs or severe malaria on Days 0-3 in the presence of parasitemia - Parasitemia on Day 2 higher than Day 0 count irrespective of temperature - Temperature > 37.5°C (Axillary) on Day 3 in presence of parasitemia - Parasitemia on Day 3 > 25% Day 0 count |
| LTF (Late Treatment Failure): Days 4 To 28  - Development of danger signs or severe malaria on Days 4 to 28 in the presence of parasitemia, without previously meeting any of the criteria of ETF. - Temperature > 37.5°C (Axillary) or history of fever in last 24 hours on Days 4 to 28 in the presence of parasitemia, without previously meeting any of the criteria of ETF. |

# Parasitological Outcome Classification System

| RIII   - Severe malaria or danger signs on Day 1 or 2 with parasite count > 25% of Day 0 - Temperature > 37.5°C (Axilary) on Day 2 with parasite count > 100% of Day 0 - Parasite count on Day 3 > 25% Day 0 count (with or without fever) |
| --- |
| RII  - Severe malaria or danger signs on Day 1 or 2 with parasite density < 25% of Day 0 - Temperature > 37.5°C (A), 38.0°C (T) on Day 3 with parasite density < 25% of Day 0 - Parasitemia on Day 3 < 25% on Day 0, parasites present on Days 4-7 |
| RIParasitemia on Day 3 < 25% on Day 0, no parasites on Day 7, parasites present on Days 8-28. |
| **S** Parasitemia on Day 3 < 25% on Day 0, no parasites on Days 7-28 |

**New WHO guidelines for assessing response to treatment (2002)**

| ETF (Early Treatment Failure): Days 0, 1, 2, and 3 |
| --- |
| Development of danger signs or severe malaria on Days 0-3 in the presence of parasitemia |
| Parasitemia on Day 2 higher than on Day 0, irrespective of temperature |
| Parasitemia on Day 3 with temperature > 37.5°C (axillary) |
| Parasitemia on Day 3 > 25% of count on Day 0 |
| LCF (Late Clinical Failure): Days 4 To 28 |
| Development of danger signs or severe malaria after Day 3 in the presence of parasitemia, without previously meeting any of the criteria of early treatment failure |
| Temperature > 37.5°C (axillary), or history of fever in past 24 hours, on Days 4 to 28 in the presence of parasitemia, without previously meeting any of the criteria of early treatment failure |
| LPF (Late Parasitological Failure): Days 7 To 28 |
| Presence of parasitemia on any day from Day 7 to Day 28, and temperature < 37.5°C (axillary), without previously meeting any of the criteria of early or late treatment failure |
| **ACPR (Adequate Clinical and Parasitological Response)** |
| Absence of parasitemia on Day 28, irrespective of temperature, without previously meeting any of the criteria of early or late treatment failure |

### APPENDIX 4. WEIGHT-BASED ADMINISTRATION OF STUDY MEDICATIONS

**Weight-Based Administration of Study Medications (2-arm study)**

| **Weight (kg)** | **Amodiaquine (AQ)**  **200 mg tabs** | | | **Chloroquine (CQ)**  **250 mg tabs** | | | **SP (500mg/**  **25 mg)** |
| --- | --- | --- | --- | --- | --- | --- | --- |
|  | **Day 0** | **Day 1** | **Day 2** | **Day 0** | **Day 1** | **Day 2** | **Day 0** |
| 5 | ¼ | ¼ | ¼ | ¼ | ¼ | ¼ | ¼ |
| 6 | ¼ | ¼ | ¼ | ½ | ¼ | ¼ | ¼ |
| 7 | ¼ | ¼ | ¼ | ½ | ½ | ¼ | ¼ |
| 8 | ½ | ¼ | ¼ | ½ | ½ | ½ | ½ |
| 9 | ½ | ¼ | ¼ | ½ | ½ | ½ | ½ |
| 10 | ½ | ½ | ¼ | ¾ | ½ | ½ | ½ |
| 11 | ½ | ½ | ¼ | ¾ | ¾ | ½ | ½ |
| 12 | ½ | ½ | ½ | ¾ | ¾ | ½ | ½ |
| 13 | ½ | ½ | ½ | 1 | ¾ | ½ | ¾ |
| 14 | ¾ | ½ | ½ | 1 | 1 | ½ | ¾ |
| 15 | ¾ | ½ | ½ | 1 | 1 | ½ | ¾ |
| 16 | ¾ | ¾ | ½ | 1 | 1 | ¾ | ¾ |
| 17 | ¾ | ¾ | ½ | 1 | 1 | 1 | ¾ |
| 18 | 1 | ¾ | ½ | 1 | 1 | 1 | ¾ |
| 19 | 1 | ¾ | ½ | 1 ¼ | 1 | 1 | 1 |
| 20 | 1 | 1 | ½ | 1 ¼ | 1 ¼ | 1 | 1 |
| 21 | 1 | 1 | ½ | 1 ¼ | 1 ¼ | 1 | 1 |
| 22 | 1 ¼ | 1 | ½ | 1 ½ | 1 ½ | ¾ | 1 |
| 23 | 1 ¼ | 1 | ½ | 1 ½ | 1 ½ | 1 | 1 ¼ |
| 24 | 1 ¼ | 1 ¼ | ½ | 1 ½ | 1 ½ | 1 | 1 ¼ |
| 25 | 1 ¼ | 1 ¼ | ½ | 1 ½ | 1 ½ | 1 ¼ | 1 ¼ |
| 26-27 | 1 ¼ | 1 | 1 | 1 ½ | 1 ½ | 1 ¼ | 1 ½ |
| 28 | 1 ¼ | 1 ¼ | 1 | 2 | 1 ½ | 1 ½ | 1 ½ |
| 29-31 | 1 ¼ | 1 ¼ | 1 | 2 | 2 | 1 | 1 ½ |
| 32 | 1 ½ | 1 ½ | 1 | 2 | 2 | 1 ½ | 1 ½ |
| 33 | 1 ½ | 1 ½ | 1 | 2 | 2 | 1 ½ | 1 ½ |
| 34-35 | 1 ½ | 1 ½ | 1 ¼ | 2 | 2 | 2 | 1 ½ |
| 36-37 | 1 ½ | 1 ½ | 1 ¼ | 2 | 2 | 2 | 1 ½ |
| 38 | 2 | 1 ½ | 1 ¼ | 2 | 2 | 2 | 2 |
| 39 | 2 | 1 ½ | 1 ¼ | 2 | 2 | 2 | 2 |
| 40-43 | 2 | 2 | 1 | 2 ½ | 2 ½ | 1 ½ | 2 |
| 44-47 | 2 | 2 | 1 ½ | 3 | 3 | 2 | 2 ½ |
| 48-49 | 2 | 2 | 2 | 3 | 3 | 2 | 2 ½ |
| 50-59 | 2 ½ | 2 | 2 | 4 | 4 | 2 | 2 ¾ |
| >60 | 3 | 3 | 1 ½ | 4 | 4 | 2 | 3 |

**Weight-Based Administration of Study Medications (3-arm study)**

| **Weight (kg)** | **Amodiaquine (AQ)**  **200 mg tabs** | | | **Artesunate (AS)**  **50 mg tabs** | | | **Chloroquine (CQ)**  **250 mg tabs** | | | **SP** | | |
| --- | --- | --- | --- | --- | --- | --- | --- | --- | --- | --- | --- | --- |
|  |  |  |  |  |  |  |  |  |  | **SP (500mg/ 25mg)** | **Placebo** | |
|  | **Day 0** | **Day 1** | **Day 2** | **Day 0** | **Day 1** | **Day 2** | **Day 0** | **Day 1** | **Day 2** | **Day 0** | **Day 1** | **Day 2** |
| 5 | ¼ | ¼ | ¼ | ½ | ½ | ½ | ¼ | ¼ | ¼ | ¼ | ½ | ½ |
| 6 | ¼ | ¼ | ¼ | ½ | ½ | ½ | ½ | ¼ | ¼ | ¼ | ½ | ½ |
| 7 | ¼ | ¼ | ¼ | ½ | ½ | ½ | ½ | ½ | ¼ | ¼ | ½ | ½ |
| 8 | ½ | ¼ | ¼ | ¾ | ¾ | ¾ | ½ | ½ | ½ | ½ | ¾ | ¾ |
| 9 | ½ | ¼ | ¼ | ¾ | ¾ | ¾ | ½ | ½ | ½ | ½ | ¾ | ¾ |
| 10 | ½ | ½ | ¼ | ¾ | ¾ | ¾ | ¾ | ½ | ½ | ½ | ¾ | ¾ |
| 11 | ½ | ½ | ¼ | 1 | 1 | 1 | ¾ | ¾ | ½ | ½ | 1 | 1 |
| 12 | ½ | ½ | ½ | 1 | 1 | 1 | ¾ | ¾ | ½ | ½ | 1 | 1 |
| 13 | ½ | ½ | ½ | 1 | 1 | 1 | 1 | ¾ | ½ | ¾ | 1 | 1 |
| 14 | ¾ | ½ | ½ | 1 ¼ | 1 ¼ | 1 ¼ | 1 | 1 | ½ | ¾ | 1 ¼ | 1 ¼ |
| 15 | ¾ | ½ | ½ | 1 ¼ | 1 ¼ | 1 ¼ | 1 | 1 | ½ | ¾ | 1 ¼ | 1 ¼ |
| 16 | ¾ | ¾ | ½ | 1 ¼ | 1 ¼ | 1 ¼ | 1 | 1 | ¾ | ¾ | 1 ¼ | 1 ¼ |
| 17 | ¾ | ¾ | ½ | 1 ½ | 1 ½ | 1 ½ | 1 | 1 | 1 | ¾ | 1 ½ | 1 ½ |
| 18 | 1 | ¾ | ½ | 1 ½ | 1 ½ | 1 ½ | 1 | 1 | 1 | ¾ | 1 ½ | 1 ½ |
| 19 | 1 | ¾ | ½ | 1 ½ | 1 ½ | 1 ½ | 1 ¼ | 1 | 1 | 1 | 1 ½ | 1 ½ |
| 20 | 1 | 1 | ½ | 1 ¾ | 1 ¾ | 1 ¾ | 1 ¼ | 1 ¼ | 1 | 1 | 1 ¾ | 1 ¾ |
| 21 | 1 | 1 | ½ | 1 ¾ | 1 ¾ | 1 ¾ | 1 ¼ | 1 ¼ | 1 | 1 | 1 ¾ | 1 ¾ |
| 22 | 1 ¼ | 1 | ½ | 1 ¾ | 1 ¾ | 1 ¾ | 1 ½ | 1 ½ | ¾ | 1 | 1 ¾ | 1 ¾ |
| 23 | 1 ¼ | 1 | ½ | 2 | 2 | 2 | 1 ½ | 1 ½ | 1 | 1 ¼ | 2 | 2 |
| 24 | 1 ¼ | 1 ¼ | ½ | 2 | 2 | 2 | 1 ½ | 1 ½ | 1 | 1 ¼ | 2 | 2 |
| 25 | 1 ¼ | 1 ¼ | ½ | 2 | 2 | 2 | 1 ½ | 1 ½ | 1 ¼ | 1 ¼ | 2 | 2 |
| 26-27 | 1 ¼ | 1 | 1 | 2 ¼ | 2 ¼ | 2 ¼ | 1 ½ | 1 ½ | 1 ¼ | 1 ½ | 2 ¼ | 2 ¼ |
| 28 | 1 ¼ | 1 ¼ | 1 | 2 ¼ | 2 ¼ | 2 ¼ | 2 | 1 ½ | 1 ½ | 1 ½ | 2 ¼ | 2 ¼ |
| 29-31 | 1 ¼ | 1 ¼ | 1 | 2 ½ | 2 ½ | 2 ½ | 2 | 2 | 1 | 1 ½ | 2 ½ | 2 ½ |
| 32 | 1 ½ | 1 ½ | 1 | 2 ½ | 2 ½ | 2 ½ | 2 | 2 | 1 ½ | 1 ½ | 2 ½ | 2 ½ |
| 33 | 1 ½ | 1 ½ | 1 | 2 ¾ | 2 ¾ | 2 ¾ | 2 | 2 | 1 ½ | 1 ½ | 2 ¾ | 2 ¾ |
| 34-35 | 1 ½ | 1 ½ | 1 ¼ | 2 ¾ | 2 ¾ | 2 ¾ | 2 | 2 | 2 | 1 ½ | 2 ¾ | 2 ¾ |
| 36-37 | 1 ½ | 1 ½ | 1 ¼ | 3 | 3 | 3 | 2 | 2 | 2 | 1 ½ | 3 | 3 |
| 38 | 2 | 1 ½ | 1 ¼ | 3 | 3 | 3 | 2 | 2 | 2 | 2 | 3 | 3 |
| 39 | 2 | 1 ½ | 1 ¼ | 3 ¼ | 3 ¼ | 3 ¼ | 2 | 2 | 2 | 2 | 3 ¼ | 3 ¼ |
| 40-43 | 2 | 2 | 1 | 3 ¼ | 3 ¼ | 3 ¼ | 2 ½ | 2 ½ | 1 ½ | 2 | 3 ¼ | 3 ¼ |
| 44-47 | 2 | 2 | 1 ½ | 3 ½ | 3 ½ | 3 ½ | 3 | 3 | 2 | 2 ½ | 3 ½ | 3 ½ |
| 48-49 | 2 | 2 | 2 | 3 ¾ | 3 ¾ | 3 ¾ | 3 | 3 | 2 | 2 ½ | 3 ¾ | 3 ¾ |
| 50-59 | 2 ½ | 2 | 2 | 4 | 4 | 4 | 4 | 4 | 2 | 2 ¾ | 4 | 4 |
| >60 | 3 | 3 | 1 ½ | 5 | 5 | 5 | 4 | 4 | 2 | 3 | 5 | 5 |

**Appendix 5. Guidelines for Grading Patient Symptoms**

|  | **Grade 1**  **MILD** | **Grade 2**  **MODERATE** | **Grade 3**  **SEVERE** | **Grade 4**  **LIFE THREATENING** |
| --- | --- | --- | --- | --- |
| **Subjective fever in the past 24 h** | N/A | Present (Yes) | N/A | N/A |
| **Convulsion** | N/A | N/A | Localized or generalized seizure | Status epilepticus |
| **Headache*** | Mild, no therapy required | Transient, moderate; therapy required | Severe; responds to initial narcotic therapy | Intractable; requires repeated narcotic therapy |
| **Anorexia** | Decreased appetite, but still taking solid food | Decreased appetite, avoiding solid food | Refusing to breast feed, appetite very decreased, no solids or liquids taken (< 2 years < 12 hr; > 2 years < 24 hr) | Refusing to breast feed, appetite very decreased, no solids or liquids taken (< 2 years < 12 hr; > 2 years < 24 hr) |
| **Nausea*** | Mild discomfort; maintains reasonable intake | Moderate discomfort; intake decreased significantly; some activity limited | Severe discomfort; no significant intake; activities limited | Minimal fluid intake |
| **Vomiting** | Transient emesis | Occasional or moderate vomiting | Orthostatic hypotension or IV fluids required | Hypotensive shock or hospitalization required for IV fluid therapy |
| **Abdominal pain*** | Mild | Moderate – no treatment needed | Moderate to severe – treatment needed | Severe – hospitalized for treatment |
| **Diarrhea** | Transient 3-4 loose stools/day | 5-7 loose stools/day | Orthostatic hypotension or > 7 loose stools/day or IV fluids required | Hypotensive shock or hospitalization for IV fluid therapy required |
| **Cough** | Transient – no treatment required | Continuous, requires treatment | Uncontrolled | Cyanosis, stridor, severe shortness of breath |
| **Pruritis** | Pruritis without rash | Pruritic rash, pruritis without rash that disturbs sleep | Mild urticaria | Severe urticaria, anaphylaxis, angioedema |
| **Tinnitus*** | Mild ringing or roaring sound | Moderate ringing or roaring sound | Severe ringing or roaring sound with associated hearing loss | N/A |
| **Behavioural changes** | Mild difficulty concentrating; mild confusion or agitation; activities of daily living unaffected; no treatment | Moderate confusion or agitation; some limitation of activities of daily living; minimal treatment | Severe confusion or agitation; Needs assistance for activities of daily living; therapy required | Toxic psychosis; hospitalization required |
| **“Flu”**  **(viral URI)** | Mild nasal congestion, mild rhinorrhea, no cough | Moderate nasal congestion, moderate rhinorrhea, cough present | N/A (if severe, classify individual symptoms) | N/A (if life-threatening, classify individual symptoms) |
| *** Assess only in children > 3 years of age. Answer N/A for younger children and those unable to answer.** | | | | |

###### † Reference – Based on WHO Toxicity Grading Scale for Determining the Severity of Adverse Events

###### APPENDIX 6. Guidelines for Grading Examination Findings

###### Table A. Guidelines for Physical Examination

| **Dehydration** | Assess skin touch and turgor, mucous membranes, eyes, crying, fontanelle, pulse, urine output |
| --- | --- |
| **Facial edema** | Assess for swelling of eyes, face, mouth |
| **Jaundice** | Assess for yellowing of the sclera. Also evaluate the palpepral conjunctiva, lips, and skin. |
| **Chest** | Observe the rate, rhythm, depth, and effort of breathing. Check the patient’s colour for cyanosis.  The maximum acceptable respiratory rate by age: **< 2 months = 60, 2-12 months = 50, 1-5 years = 40, above 5 years = 30.**  Inspect the neck for the position of the trachea, for supraclavicular retractions, and for contraction of the sternomastoid or other accessory muscles during inspiration.  Auscultate the anterior and posterior chest for normal breath sounds and any adventitious sounds (crackles or rales, wheezes, and rhonchi). *Crackles are intermittent, non-musical, fine or coarse sounds that may be due to abnormalities of the lungs (pneumonia, fibrosis, early congestive heart failure) or airways (bronchitis or bronchiectasis). Wheezes are high-pitched and result from narrowed airways. Rhonchi are relatively low-pitched and suggest secretions in large airways.*  If abnormalities are identified, evaluate for transmitted voice sounds. In addition, palpate the chest to assess for tactile fremitus, and percuss the chest to assess for areas of dullness*. Normal, air-filled lungs emit predominantly vesicular breath sounds, transmit voice sounds poorly with “ee” = “ee”, and have no tactile fremitus. Airless lung, as in lobar pneumonia, emits bronchial breath sounds, transmits spoken words clearly with “ee” = “aay” (egophany), and has an increase in tactile fremitus.* |
| **Abdomen** | Inspect and ausculate the abdomen. Listen for bowel sounds in the abdomen before palpating it. Palpate the abdomen in all 4 quadrants lightly and then deeply. Assess the size of the liver and spleen. To assess for peritoneal inflammation, look for localised and rebound tenderness, and voluntary or involuntary rigidity. |
| **Skin** | Inspect the skin for colour, turgor, moisture, and lesions. If lesions are present, note their location and distribution (diffuse or localised), arrangement (linear, clustered, annular, dermatomal), type (macules, papules, vesicles) and colour. |
| **Hearing** | In children < 4 years, test hearing by shaking a rattle or crinkling paper from behind the head of the child on the right and left. Note child’s reaction to the noise – inspecting for a startle, or turning of the head toward the direction of the noise.  In children > 4 years of age, estimate hearing by testing one ear at a time. Ask the patient to occlude one ear with a finger or, better still, occlude it yourself. Gently rub your thumb and index finger together within 2 inches or 5 cm from the patient’s unoccluded ear and assess if the patient is able to hear the noise.  In children of all ages, perform a full otoscopic exam if these screening methods reveal any diminunition of hearing. In patients > 4 years of age only, attempt to assess for conductive and sensorineural hearing loss, using the tuning fork. Set the fork into light vibration by tapping it. Place the base of the lightly vibrating tuning fork firmly on top of the patient’s head or midforehead and test for lateralization (Weber test). Ask where the patient hears it: on one or both sides. Normally, the sound is heard in the midline or equally in both ears. If nothing is heard, try again, pressing the fork more firmly on the head. In unilateral conductive hearing loss, sound is heard in (lateralized to) the impaired ear. Visible explanations include acute otitis media, perforation of the eardrum, and obstruction of the ear canal, as by cerumen. In unilateral sensorineural hearing loss, sound is heard in the good ear.  Next, to compare air conduction (AC) and bone conduction (BC) perform the Rinne test. Place the base of lightly vibrating tuning fork on the mastoid bone, behind the ear and level with the canal. When the patient can no longer hear the sound, quickly place the fork close to the ear canal and ascertain whether the sound can be heard again. Hear the “U” of the fork should face forward, thus maximising its sound for the patient*. Normally, the sound is heard longer through air than through bone (AC>BC). In conductive hearing loss, sound is heard through bone as long as or longer than it is through air (BC=AC or BC>AC). In sensorineural hearing loss, sound is heard longer through air (AC>BC).* |
| **Nystagmus** | Assess the extraocular movements in children of all ages by moving a torch or brightly coloured object in a circle, looking for conjugate movements of the eyes in each directions, or any deviation from normal and for nystagmus, a fine rhythmic oscillation of the eyes, analogous to a tremor in other parts of the body. *A few beats of nystagmus on extreme lateral gaze are within normal limits. If you see it, bring your finger in to within the field of binocular vision and look again*.  *The causes of nystagmus are multiple, including impairment of vision in early life, disorders of the labyrinth and the cerebellar system, and drug toxicity. Nystagmus occurs normally when a person watches a rapidly moving object. Nystagmus usually has both fast and slow movements, but is defined by its fast phase. For example, if the eyes jerk quickly to the patient’s left and drift back slowly to the right, the patient is said to have nystagmus to the left. The movements of nystagmus may occur in one or more planes (i.e. horizontal, vertical, or rotatory). It is the plane of the movements, not the direction of the gaze that defines this variable.* |
| **Tablet test** | For children > 9 months of age, ask the patient to pick a tablet (or equivalent object) up off a flat surface using the thumb and index finger of their dominant hand*. This tests for co-ordination of the upper extremity assessing the function of the motor system, cerebellar system, vestibular system (for coordinating eye and body movements) and the sensory system, for position sense. When testing small children, be aware that they will likely attempt to put the object into their mouth.* |
| **Heel-toe** | For patients > 4 years of age, asks the patient to walk heel-to-toe in a straight line – a pattern called tandem walking. If the patient is unable to perform this, ask them to walk across the room normally, and/or to walk on their toes and then on the heels, to further assess gait pattern. A gait that lacks coordination in instability is called ataxic and may be due to cerebellar disease or loss of position sense.  For children 2-4 years of age, assess gait by having them walk in a straight line. |
| **Romberg** | This should be performed in children > 4 years and is mainly a test of position sense. The patient should first stand with feet together and eyes open and then close both eyes for 20 to 30 seconds without support. Note the patient’s ability to maintain an upright posture. To test for pronator drift, have the patient stand for 20 to 30 seconds with both arms straightforward, palms up, and with eyes closed. A person who cannot stand may be tested for pronator drift in the sitting position. In either case, a normal person can hold this arm position well. Now, instructing the patient to keep the arms up and eyes shut, tap the arms briskly downward. The arms normally return smoothly to the horizontal position. This response requires muscular strength, coordination, and a good sense of position.  In ataxia due to loss of position sense, vision compensates for the sensory loss. The patient stands fairly well with eyes open, but loses balance when they are closed, a **positive Romberg sign**. In cerebellar ataxia, the patient has difficulty standing with feet together whether the eyes are open or closed. |

**Table B. Grading Physical Examination Findings**

|  | **Grade 1**  **MILD** | **Grade 2**  **MODERATE** | **Grade 3**  **SEVERE** | **Grade 4**  **LIFE-THREATENING** |
| --- | --- | --- | --- | --- |
| **Dehydration*** | Normal skin turgor and touch, moist mucous membranes, tears present, eyes normal, fontanelle flat, CNS – consolable, pulse regular, urine output normal | Skin dry with + tenting, dry mucous membranes, eyes deep set, decreased tears, fontanelle soft, CNS – irritable, pulse slightly increased, urine output decreased | Skin clammy with lack of turgor, parched / cracked mucous membranes, sunken eyes, no tears, sunken fontanelle, CNS – lethargic, pusle increased, no urine output | |
| **Temperature* (Axillary)** | 37.5-37.9°C | 38.0-39.5°C | > 39.5°C | Sustained fever, equal or greater than 40.0°C for longer than 5 days |
| **Jaundice** | Slight yellowing of sclera and conjunctiva | Moderate yellowing of sclera and conjunctiva, yellowing of mucous membranes | Severe yellowing of sclera and conjunctiva, yellowing of skin | N/A |
| **Chest** | Mildly increased RR (for age, temperature), transient or localised adventitious sounds | Moderately increased RR, diffuse or persistent adventitious sounds | Rapid RR (< 2 months > 60, 2-12 months > 50, 1-5 years > 40, adults > 30)*nasal flaring, retractions | Cyanosis |
| **Abdomen** | Normal bowel sounds, mild localised tenderness, and/or liver palpable 2-4 cm below the right costal margin (RCM), and/or spleen palpable, and/or umbilical hernia present | Normal or mildly abnormal bowel sounds, moderate or diffuse tenderness; and/or mild to moderately enlarged liver (4-6 cm below the RCM) and/or spleen palpable up to half-way between umbilicus and symphysis pubis | Severely abnormal bowel sounds, severe tenderness to palpation. Evidence of peritoneal irritation and/or significant enlargement of liver (> 6 cm below the RCM) and/or spleen palpable beyond half-way between umbilicus and symphysis pubis | Absent bowel sounds. Involuntary rigidity |
| **Skin†** | Localised rash, erythema, or pruritis | Diffuse, maculopapular rash, dry desquamation | Vesiculation, moist desquamation, or ulceration | Exfoliative dermatitis, mucous membrane involvement or erythema multiforme or suspected Stevens-Johnson or necrosis requiring surgery |

* Reference – The Harriet Lane Handbook, 15^th^ edition, 2000

† Reference – WHO Toxicity Grading Scale for Determining the Severity of Adverse Events

|  | **Grade 1**  **MILD** | **Grade 2**  **MODERATE** | **Grade 3**  **SEVERE** | **Grade 4**  **LIFE-THREATENING** |
| --- | --- | --- | --- | --- |
| **Hearing** | *< 4 years: N/A*  > 4 years: Decreased hearing in one ear | *< 4 years: N/A*  > 4 years: Decreased hearing in both ears or severe impairment in one ear | *< 4 years: Any evidence of hearing impairment*  > 4 years: Severe impairment in both ears | N/A |
| **Nystagmus** | 3 or fewer beats of lateral nystagmus | More than 3 beats of lateral nystagmus | Sustained lateral nystagmus, any vertical or rotary nystagmus | N/A |
| **Tablet test** | Difficulty grasping tablet but able to pick up | Unable to pick up tablet without dropping | Unable to grasp tablet | N/A |
| **Heel-toe** | 2-4 years: Able to take at least 5 steps  > 4 years: Able to take at least 5 tandem steps | 2-4 years: Unable to take 5 steps  > 4 years: Unable to take 5 tandem steps | *2-4 years: Unable to walk*  > 4 years: Unable to perform tandem walk | N/A |
| **Romberg** | > 4 years: Unable to stand for 30 seconds with eyes closed | > 4 years: Unable to stand for 15 seconds with eyes closed | > 4 years: Unable to stand with feet together with eyes open or closed | N/A |
| **Clinical symptoms / sign** *(not otherwise specified)* | No therapy; monitor condition | May require minimal intervention and monitoring | Requires medical care and possible hospitalization | Requires active medical intervention, hospitalization, or hospice care |

**TABLE C. Guidelines for Grading of Laboratory Abnormalities**

|  | **Grade 1**  **MILD** | **Grade 2**  **MODERATE** | **Grade 3**  **SEVERE** | **Grade 4**  **LIFE-THREATENING** |
| --- | --- | --- | --- | --- |
| **Hemoglobin**  ***(****g/dL)* | 9.5-10.5 g/dL | 8.0-94 g/dL | 6.5-7.9 g/Dl | < 6.5 g/dL |
| **Laboratory values** *(not otherwise specified)* | Abnormal but requiring no immediate intervention; follow | Sufficiently abnormal to require evaluation as to causality and perhaps mild therapeutic intervention | Sufficiently severe to require evaluation and treatment | Life-threatening severity; requires immediate evaluation, treatment, and usually hospitalization |

*Reference – WHO Toxicity Grading Scale for Determining the Severity of Adverse Events

**Appendix 7. Informed Consent**

**Study number:__________________________**

### CONSENT TO PARTICIPATE IN A RESEARCH STUDY

## Title of Study: Combination therapies for treatment of uncomplicated malaria in Uganda: evaluation of efficacy, safety, and tolerability.

**2-Arm Study**

**A. Purpose and Background**

Dr. Fred Wabwire-Mangen of Makerere University and Dr. Arthur Reingold of University of California, Berkeley, and Drs. Moses Kamya and Philip Rosenthal of the Makerere University – University of California, San Francisco (MU-UCSF) Malaria Research Collaboration are doing a study to learn more about malaria and the response of this illness to drugs. The study is funded by the United States Centers for Disease Control (CDC) and conducted by the Uganda Malaria Surveillance Project (UMSP), which is a collaboration between Makerere University Institute of Public Health, MU-UCSF Malaria Research Collaboration, the Ugandan Ministry of Health and UC Berkeley. We would like to know what the safest and best treatment for uncomplicated malaria is in Uganda. To do this, we are carrying out a research study in which patients are treated for malaria with combinations of antimalarial drugs including: chloroquine plus sulfadoxine/pyrimethamine (CQ/SP) and amodiaquine plus sulfadoxine/pyrimethamine (AQ/SP). Following treatment, participants will be followed for 28 days to see if their infection is cured. About 360 patients with malaria will be involved in this study.

The choice of drugs that you or your child will receive will be determined by a process of randomization. Randomization means that you or your child will be put into a group by chance (similar to pulling a number out of a hat). You will not be told which treatment you or your child has been assigned to receive. You are being asked to allow yourself or your child (or the child under your care in the case of a legal guardian) to participate in this study for up to 28 days or until such time as you or the study doctors decide that you or your child should no longer participate in the study.

## B. Procedures:

## A physical examination will be performed.

## Blood will be collected. A small amount of blood will be taken by finger-prick to examine for malaria parasites, to measure hemoglobin, to store blood samples on filter paper for future laboratory tests, and to store for future research purposes.

1. If the diagnosis of malaria is confirmed, and you or your child is eligible for the study, he/she will be treated with chloroquine + SP or amodiaquine + SP. All of these drugs are currently registered for the treatment of malaria in Uganda.
2. You will be asked to return to the clinic 7 more times over the next 4 weeks so that the success of the treatment can be judged. At each of the follow-up visits, you or your child will have a physical examination and for 5 of these visits a small amount of blood will taken by fingerprick to examine for malaria parasites and to save on filter paper. Hemoglobin will be measured again on the last day.
3. If you or your child misses an appointment, the home health visitor will visit you at your home to find out why you missed the appointment and bring you or your child to the clinic for assessment.
4. If, at any time, the treatment given to you or your child does not seem to be working well, it will be changed to quinine.
5. There will be someone at the study clinic every day from 8:00 am to 5:00 pm. You/your child should come/be brought to the clinic to be evaluated anytime that you/your child feels ill during the next 28 days.
6. During the study, you will be informed promptly of any new information that may influence your willingness to continue participation in the study.

## C. Risks/Discomforts

1. You or your child will be assigned to a treatment program by chance. The treatment you or your child receives may prove to be less effective or to have more side effects than the other study treatments or than other available treatments. This will not be known until after the study is completed and the data has been analysed.
2. Undesirable side effects have rarely been reported with the use of both amodiaquine (Camoquin) and SP (Fansidar) including skin rashes, lowering of blood counts, inflammation of the liver, and death. You or your child will be monitored closely after receiving treatment for malaria with the study medications for any possible side effects of the drugs and will receive appropriate medical care for any problem arising during the course of the study.
3. Having blood drawn may cause some discomfort, bleeding, or bruising where the lancet enters the body, and rarely may cause fainting or infection. The amount of blood removed will be too small to affect you or your child’s health.
4. If you or your child is injured as a result of the study, treatment will be available and the cost of treatment will be paid by UMSP.
5. Participation in research may cause a loss of privacy, but information about you will be kept as confidential as possible. Representatives from the CDC may review information about you to check on the study. Your name will not be used in any published reports of this study.

**D. Benefits**

The potential benefit to you or your child is that the treatment you or your child receives may prove to be more effective than the other study treatments or than other available treatments, although this cannot be guaranteed. The knowledge gained from this study will also help the country of Uganda in determining the best treatment for uncomplicated malaria.

**E. Alternatives**

You or your child’s participation in this study is completely voluntary. If you choose not to participate, there will be no effect on you or your child’s ability to receive standard treatment for malaria. You may withdraw yourself or your child from the study at any time and for any reason. If you choose not to participate in the study, you or your child can receive treatment in the Outpatient Department.

**F. Cost/Payment**

After enrollment in the study, you will not be charged for clinic visits or treatment. You or your child will not be paid for participation in the study. You will be reimbursed for transport costs to and from the clinic for any visit that you or your child requires.

**G. Questions**

The Medical Officer in charged will explain this study to you. If you have any questions you may ask them now or any time during the study. You may ask to speak with Dr. Kamya (telephone 041-541188 or 041-533200) at Mulago Hospital, Kampala or Dr. Nathan Bakyaita (telephone 077-601579) at the Ugandan Ministry of Health.

**H. Consent**

A copy of this consent form will be provided for you to keep at your request. You or your child’s participation in this research study is completely voluntary. No effect on your ability to receive care at the clinic will result if you or your child does not participate in this study. You or your child may be withdrawn from the study at any time that you, or the investigators, choose to do so, and you or your child can then receive a standard treatment for malaria. By signing this form, you are telling the study staff that you are willing to join the research project described above. If you agree to participate or let your child participate, you should sign below.

Name of Participant (printed)

Signature or Fingerprint * of participant over 18 years Date/Time

**For children / adolescents:**

Name of Parent/Guardian

Signature or Fingerprint * of Parent/Guardian Date/Time

**For adolescents aged 12-18 years:**

If the patient is a minor aged 12-18 years, has participated in and understood the informed consent process, and assents to participate in the study, he/she should sign below:

Signature or Fingerprint * of participant aged 12-18 years Date/Time

Name of Investigator Administering Consent (printed) Position/Title

Signature of Investigator Administering Consent Date/Time

*If the patient, parent or guardian is unable to read and/or write, an impartial witness should be present during the informed consent discussion. After the written informed consent form is read and explained to the participant, parent or guardian, and after they have orally consented to their or their child’s participation in the trial, and have either signed the consent form or provided their fingerprint, the witness should sign and personally date the consent form. By signing the consent form, the witness attests that the information in the consent form and any other written information was accurately explained to, and apparently understood by, the patient, parent or guardian, and that informed consent was freely given by the patient, parent or guardian.

Name of Person Witnessing Consent (printed)

Signature of Person Witnessing Consent Date/Time

**Study number:__________________________**

### CONSENT TO PARTICIPATE IN A RESEARCH STUDY

## Title of Study: Combination therapies for treatment of uncomplicated malaria in Uganda: evaluation of efficacy, safety, and tolerability.

**3-Arm Study**

**A. Purpose and Background**

Dr. Fred Wabwire-Mangen of Makerere University, Dr. Arthur Reingold of University of California, Berkeley, and Drs. Moses Kamya and Philip Rosenthal of the Makerere University – University of California, San Francisco (MU-UCSF) Malaria Research Collaboration are doing a study to learn more about malaria and the response of this illness to drugs. The study is funded by the United States Centers for Disease Control (CDC) and conducted by the Uganda Malaria Surveillance Project (UMSP), which is a collaboration between Makerere University Institute of Public Health, MU-UCSF Malaria Research Collaboration, the Ugandan Ministry of Health and UC Berkeley. We would like to know what the safest and best treatment for uncomplicated malaria is in Uganda. To do this, we are carrying out a research study in which patients are treated for malaria with combinations of antimalarial drugs including: chloroquine plus sulfadoxine/pyrimethamine (CQ/SP), amodiaquine plus sulfadoxine/pyrimethamine (AQ/SP), and amodiaquine plus artesunate (AQ/AS). Following treatment, participants will be followed for 28 days to see if their infection is cured. About 540 patients with malaria will be involved in this study.

The choice of drugs that you or your child will receive will be determined by a process of randomization. Randomization means that you or your child will be put into a group by chance (similar to pulling a number out of a hat). You will not be told which treatment you or your child has been assigned to receive. You are being asked to allow yourself or your child (or the child under your care in the case of a legal guardian) to participate in this study for up to 28 days or until such time as you or the study doctors decide that you or your child should no longer participate in the study.

## B. Procedures:

## A physical examination will be performed.

## Blood will be collected. A small amount of blood will be taken by finger-prick to examine for malaria parasites, to measure hemoglobin, to store blood samples on filter paper for future laboratory tests, and to store for future research purposes.

1. If the diagnosis of malaria is confirmed, and you or your child is eligible for the study, he/she will be treated with chloroquine + SP, amodiaquine + SP, or amodiaquine + artesunate. All of these drugs are currently registered for the treatment of malaria in Uganda.
2. You will be asked to return to the clinic 7 more times over the next 4 weeks so that the success of the treatment can be judged. At each of the follow-up visits, you or your child will have a physical examination and for 5 of these visits a small amount of blood will taken by fingerprick to examine for malaria parasites and to save on filter paper. Hemoglobin will be measured again on the last day.
3. If you or your child misses an appointment, the home health visitor will visit you at your home to find out why you missed the appointment and bring you or your child to the clinic for assessment.
4. If, at any time, the treatment given to you or your child does not seem to be working well, it will be changed to quinine.
5. There will be someone at the study clinic every day from 8:00 am to 5:00 pm. You/your child should come/be brought to the clinic to be evaluated anytime that you/your child feels ill during the next 28 days.
6. During the study, you will be informed promptly of any new information that may influence your willingness to continue participation in the study.

## C. Risks/Discomforts

1. You or your child will be assigned to a treatment program by chance. The treatment you or your child receives may prove to be less effective or to have more side effects than the other study treatments or than other available treatments. This will not be known until after the study is completed and the data has been analysed.
2. Undesirable side effects have rarely been reported with the use of both amodiaquine (Camoquin) and SP (Fansidar) including skin rashes, lowering of blood counts, inflammation of the liver, and death. Major side effects appear to be extremely rare in people treated with artesunate. You or your child will be monitored closely after receiving treatment for malaria with the study medications for any possible side effects of the drugs and will receive appropriate medical care for any problem arising during the course of the study.
3. Having blood drawn may cause some discomfort, bleeding, or bruising where the lancet enters the body, and rarely may cause fainting or infection. The amount of blood removed will be too small to affect you or your child’s health.
4. If you or your child is injured as a result of the study, treatment will be available and the cost of treatment will be paid by UMSP.
5. Participation in research may cause a loss of privacy, but information about you will be kept as confidential as possible. Representatives from the CDC may review information about you to check on the study. Your name will not be used in any published reports of this study.

**D. Benefits**

The potential benefit to you or your child is that the treatment you or your child receives may prove to be more effective than the other study treatments or than other available treatments, although this cannot be guaranteed. The knowledge gained from this study will also help the country of Uganda in determining the best treatment for uncomplicated malaria.

**E. Alternatives**

You or your child’s participation in this study is completely voluntary. If you choose not to participate, there will be no effect on you or your child’s ability to receive standard treatment for malaria. You may withdraw yourself or your child from the study at any time and for any reason. If you choose not to participate in the study, you or your child can receive treatment in the Outpatient Department

**F. Cost/Payment**

After enrollment in the study, you will not be charged for clinic visits or treatment. You or your child will not be paid for participation in the study. You will be reimbursed for transport costs to and from the clinic for any visit that you or your child requires.

**G. Questions**

The Medical Officer in charged will explain this study to you. If you have any questions you may ask them now or any time during the study. You may ask to speak with Dr. Kamya (telephone 041-541188 or 041-533200) at Mulago Hospital, Kampala or Dr. Nathan Bakyaita (telephone 077-601579) at the Ugandan Ministry of Health.

**H. Consent**

A copy of this consent form will be provided for you to keep at your request. You or your child’s participation in this research study is completely voluntary. No effect on your ability to receive care at the clinic will result if you or your child does not participate in this study. You or your child may be withdrawn from the study at any time that you, or the investigators, choose to do so, and you or your child can then receive a standard treatment for malaria. By signing this form, you are telling the study staff that you are willing to join the research project described above. If you agree to participate or let your child participate, you should sign below.

Name of Participant (printed)

Signature or Fingerprint * of participant over 18 years Date/Time

**For children / adolescents:**

Name of Parent/Guardian

Signature or Fingerprint * of Parent/Guardian Date/Time

**For adolescents aged 12-18 years:**

If the patient is a minor aged 12-18 years, has participated in and understood the informed consent process, and assents to participate in the study, he/she should sign below:

Signature or Fingerprint * of participant aged 12-18 years Date/Time

Name of Investigator Administering Consent (printed) Position/Title

Signature of Investigator Administering Consent Date/Time

*If the patient, parent or guardian is unable to read and/or write, an impartial witness should be present during the informed consent discussion. After the written informed consent form is read and explained to the participant, parent or guardian, and after they have orally consented to their or their child’s participation in the trial, and have either signed the consent form or provided their fingerprint, the witness should sign and personally date the consent form. By signing the consent form, the witness attests that the information in the consent form and any other written information was accurately explained to, and apparently understood by, the patient, parent or guardian, and that informed consent was freely given by the patient, parent or guardian.

Name of Person Witnessing Consent (printed)

Signature of Person Witnessing Consent Date/Time

### Study number:_______________________

# **CONSENT FOR FUTURE USE OF BIOLOGICAL SPECIMENS**

## Title of Study: Combination therapies for treatment of uncomplicated malaria in Uganda: evaluation of safety, tolerability, and efficacy

**2 or 3-Arm Study**

**A. Introduction:** Dr. Fred Wabwire-Mangen of Makerere University and Dr. Arthur Reingold of University of California, Berkeley, and Drs. Moses Kamya and Philip Rosenthal of the Makerere University – University of California, San Francisco (MU-UCSF) Malaria Research Collaboration are doing a study to learn more about malaria and the response of this illness to drugs. The study is funded by the United States Centers for Disease Control (CDC) and conducted by the Uganda Malaria Surveillance Project (UMSP), which is collaboration between Makerere University Institute of Public Health, MU-UCSF Malaria Research Collaboration, the Ugandan Ministry of Health and UC Berkeley. We would like to know what the safest and best treatment for uncomplicated malaria is in Uganda. While you or your child is in this study, there may be blood samples taken from them that may be useful for future research. These samples will be stored at Makerere University Medical School and the University of California, San Francisco. Samples may also be shared with investigators at other institutions.

**B. What Samples Will Be Used For:** Your or your child’s blood and the malaria parasites in it will be used to study malaria and the response of this disease to treatment using genetic markers. Specific results are not likely to affect you or your child as an individual patient.

1. These samples will be used for future research to learn more about malaria and other diseases.
2. Your or your child’s samples will be used only for research and will not be sold or used directly for the production of commercial products.
3. Genetic research may be performed on samples to study the nature of malaria in people. However, no genetic information obtained from this research will be placed in yours or your child’s medical records. These samples will be identified only by codes so that they cannot be readily identified with you or your child personally.

**C. Level of Identification:**

1. Your or your child’s samples and/or cultures will be coded so that your or your child’s name cannot be readily identified. Reports about research done with your or your child’s samples will not be put in their medical record and will be kept confidential to the best of our ability within state and federal law.
2. In the future, researchers studying your or your child’s samples and/or cultures may need to know more about you or your child, such as their age, gender, and race. If this information is already available because of your or your child’s participation in a study, it may be provided to the researcher. Your or your child’s name or anything that might identify you/them personally will not be provided.

## D. Risks

There are few risks to you or your child from future use of their samples. A potential risk might be the release of information from your or your child’s health or study records. Reports about research done with your or your child’s samples will not be put in their health record, but will be kept with the study records. The study records will be kept confidential as far as possible.

## E. Benefits

There will be no direct benefit to you or your child. From studying your or your child’s samples/cultures we may learn more about malaria or other diseases: how to prevent them, how to treat them, how to cure them.

## F. Research Results/Medical Records

1. Results from future research using your or your child’s samples and/or cultures may be presented in publications and meetings but patient names will not be identified.
2. Reports from future research done with your or your child’s samples and/or cultures will not be given to you or your child’s doctor. These reports will not be put in your or your child’s medical record.

## G. Future IRB Review

Any additional research studies beyond the current study using your or your child’s identifiable samples and/or cultures will be reviewed by the investigator’s Institutional Review Board (IRB), a special committee that oversees medical research studies to protect the rights and welfare of the human subject volunteers.

## H. Freedom to Refuse

You can change your mind at any time about allowing your or your child’s identifiable samples to be used for future research. . If you do, contact Dr. Kamya (telephone 041-541188 or 041-533200) at Mulago Hospital, Kampala or Dr. Nathan Bakyaita (telephone 077-601579) at the Ugandan Ministry of Health.

Then your or your child’s samples will no longer be made available for research and will be destroyed. Whether or not you allow us to use your or your child’s identifiable samples in future research, your decision will not have any effect on you or your child’s participation in this study or future participation in other studies.

## I. Consent

A copy of this consent form will be provided for you to keep at your request. If you wish to allow your or your child’s samples and cultures to be used for future research, please sign below.

Name of Participant (printed)

Signature or Fingerprint * of participant over 18 years Date/Time

**For children / adolescents:**

Name of Parent/Guardian

Signature or Fingerprint * of Parent/Guardian Date/Time

**For adolescents aged 12-18 years:**

If the patient is a minor aged 12-18 years, has participated in and understood the informed consent process, and assents to participate in the study, he/she should sign below:

Signature or Fingerprint * of participant aged 12-18 years Date/Time

Name of Investigator Administering Consent (printed) Position/Title

Signature of Investigator Administering Consent Date/Time

*If the patient, parent or guardian is unable to read and/or write, an impartial witness should be present during the informed consent discussion. After the written informed consent form is read and explained to the patient, parent or guardian, and after they have orally consented to their or their child’s participation in the trial, and have either signed the consent form or provided their fingerprint, the witness should sign and personally date the consent form. By signing the consent form, the witness attests that the information in the consent form and any other written information was accurately explained to, and apparently understood by, the parent or guardian, and that informed consent was freely given by the patient, parent or guardian.

Name of Person Witnessing Consent (printed)

Signature of Person Witnessing Consent Date/Time

**Appendix 8: Patient Screening Form**

**STUDY SITE CODE:________ STUDY NUMBER: ___________________________**

UMSP SCREENING FORM

| **1. Names:** | **3. Date: *(dd/mm/yy)*** |
| --- | --- |
| **3. Age*: _______years __________months.** | **Gender: ______M _______F** |

**** Include months only if age < 5 years, if age is above 5 years write*** *“X”.*

| **SCREENING selection criteria**  ***Onlyscreen patients who are 6 months or older and have a positive screening thick blood smear*** | | |
| --- | --- | --- |
| **inclusion criteria** | **yes** | **NO** |
| 5. Fever (> 37.5°C) or history of fever in previous 24 h |  |  |
| 6. Ability to participate in 28 day follow up. |  |  |
| **EXCLUSION CRITERIA** | **NO** | **YES** |
| 7. History of serious side effects to study medications  (Including sulfa allergy)  *If present, indicate drug / side effect:*   Amodiaquine:________________________   Artesunate (3-arm study only):_________________   Chloroquine:_________________________   SP:_________________________________ |  |  |
| 8. Evidence of severe malaria / danger signs  *If “ YES” indicate criteria. If “NO”, leave blank.*   Unarousable coma *(if after convulsion, > 30 min)*   Repeated convulsions *(> 2 within 24 h)*   Recent convulsions *(1-2 within 24 h)*   Altered consciousness  *(confusion, delerium, psychosis, coma)*   Lethargy   Unable to drink or breast feed   Vomiting everything   Unable to stand/sit due to weakness   Severe anemia *(Hb < 5.0 g/dL)*   Respiratory distress *(laboured breathing at rest)*   Jaundice *(yellow coloring of eyes)* |  |  |
| 9. Evidence of concomitant febrile illness  *If “YES”, indicate illness. If “NO”, leave blank.*   Pneumonia/RTI  Measles   Otitis Media  Other:___________________   Gastroenteritis |  |  |
| 10. Presence of pregnancy by LNMP  N/A |  |  |
| 11. History of Anti-folate or Amodiaquine use in past week  *If “YES”, indicate drug / date taken. If “NO”, leave blank.*   AQ (> 2 days):__________  Septrin (> 3 days):_______________  SP (single dose):_ _______  Metakelfin (single dose):__________ |  |  |
| **INCLUSION CRITERIA.** | **YES** | **NO** |
| 11. Provision of informed consent. |  |  |
| ***Complete prior to day 1 clinic visit.*** | | |
| 12. *P. falciparum* infection |  |  |
| 13. Parasite density > 2000/ul and < 200,000/ul |  |  |

***If any of the responses fall into the shaded area, exclude the patient from the study***

**APPENDIX 9. UMSP ENROLLMENT FORM.**

| **enrollment form** | | |
| --- | --- | --- |
| **1. Study**  **Number:** | **2. Treatment**  **Number:** | **3. Start Date:**  ***(dd/mm/yy)*** |

| 4.Patients name: |
| --- |
| 5. Father’s name: |
| 6. Mother’s name: |
| 7. Primary caregiver / guardian’s name and relationship (N/A if mother or father): |
| 8. Sub county of residence. |
| 9. Home parish: |
| 10. LC1/village: |
| 11. Home address and localising features: |
| 12. a) Phone number: Yes ___No ___  If yes: b) Phone number (s) and the owner(s): |
| Patient Information |
| 13. Anti-folate use in the last 4 weeks? Yes ______ No________Unknown__________ |
| 14. Does the patient use malaria preventive measures: Yes ___No ___Unknown ____  If yes, which measures are used?    a) Spray: Yes ___No ___  b) Coils: Yes ___No ___  c) Chemoprophylaxis: Yes ___No ___ 🡪 If yes, which drug? ___________________________________  e) Bed net: Yes ___No ____ 🡪 If yes, is the net treated? Yes ___No ___  🡪 How often does the patient sleep under the net?  Always____ Often / Sometimes____  h) Other protective measures ______________________________________________________________ |

### APPENDIX 10. Adverse Event Reporting Guidelines

On Day 0 perform a careful history and physical exam and record your findings on the case record form (CRF), grading the patient reported symptoms and exam findings according to the guidelines presented in Appendices 3 and 4. Record the hemoglobin value and grade (Appendix 5) on the CRF as soon as they are available. All Day 0 values will serve as the patient’s baseline.

On all follow-up days, assess and grade the patient-reported symptoms and physical exam signs according to the guidelines in Appendices 3 and 4. Record the follow-up hemoglobin results obtained on Day 28 (and any extra day) and grade according to the guidelines in Appendix 5.

At each follow-up visit, evaluate the CFR for any reportable adverse events. Review for symptoms, physical exam signs, and hemoglobin results that are > grade 2 and are new or worsening by comparing the grade assigned on the follow-up day to the previous day’s record.

If a new or worsening symptom, sign, or laboratory value is present (increasing grade compared to the previous day’s value and > grade 2), answer “YES” in the appropriate column for reporting adverse events on the CRF (otherwise, answer “NO”).

An adverse event is defined as any “untoward” (unfavorable or undesirable) medical occurrence in a patient who has received a medication that may or may not be related to this treatment. Any event (patient-reported symptom, physical exam finding, or laboratory value) that occurs after treatment with a study medication that is new or worsening from Day 0 should be considered a possible adverse event, regardless of whether you think that the treatment caused the event.

The following scenarios are examples of adverse events (AEs):

|  | **Day 0** | **Day 1** | **Day 2** |
| --- | --- | --- | --- |
| Nausea | 0 | 0 | 2 |

AE = nausea on Day 2

|  | **Day 0** | **Day 1** | **Day 2** | **Day 3** |
| --- | --- | --- | --- | --- |
| Nausea | 2 | 1 | 0 | 2 |

AE = nausea on Day 3

For each new / worsening event of > grade 2, the following information will be collected on the Adverse Event Record Form:

1. Description of event
2. Date of event onset
3. Date event reported
4. Maximum severity of the event
5. Maximum suspected relationship of the event to study medication
6. Is the event serious?
7. Initials of the person reporting the event
8. Was the event episodic or intermittent in nature?
9. Outcome
10. Date event resolved

The following definitions / guidelines should be used to complete this form:

**Description of event** = symptom, sign, laboratory abnormality

**Date of event onset** = If event occurs on Days 1-3, date of event onset is the same as date event reported. If event occurs after Day 3, estimate the date of event onset from patient history and/or data available on CRF

**Date event reported** = Date the event is first recorded on the adverse event (AE) form

**Maximum severity** = Record the grade of the event (rank on scale of 2-4: moderate = 2, severe = 3, life-threatening = 4). This column should be updated if necessary during follow-up to capture the maximum severity of the event. Changes should be made by marking through the original number with a single line, recording the new value, and placing your initials and the date the change was made next to the new result.

**Maximum suspected relationship with study medications** =

| **Classification** | **Definition** |
| --- | --- |
| Definite | Clear-cut temporal association, with laboratory confirmation, if indicated |
| Probable | Clear-cut temporal association, with improvement upon study agent withdrawal, and not reasonably explained by the subject’s known clinical state |
| Possible | Less clear temporal association, other etiologies possible |
| Unlikely | Less clear temporal association; relationship to study agent in doubt |
| None | Clearly related to other etiologies such as motor vehicle accident |

The following criteria will be assessed in order to establish the suspected relationship of the event to the study medications:

1. Timing of the onset of the event
2. New event vs. worsening of a condition present at baseline
3. Overall medical condition of the patient, including status of malaria

**Serious events** = the term “serious” depends on the outcome and/or actions taken in response to the event and is not directly related to the “severity” or intensity of the event. A serious event is one that is:

- Fatal
- Life-threatening
- Results in or prolongs hospitalization
- Requires medical or surgical interventions to prevent a serious outcome
- Results in significant or persistent disability or capacity

Any death that occurs during the study should be considered a serious AE, regardless of whether an AE had previously been reported for the patient. All serious events should be reported to the Kampala core facility staff immediately. They will assist you with management of the patient (including arranging transport of the patient to Kampala, if necessary), will be responsible for completing the serious AE report form, and will report the event to the appropriate review committees.

**Episodic or intermittent event** = At the final visit, the CRF will be reviewed in order to determine if a reported AE was episodic or intermittent in nature.

**Outcome** = Complete this on Day 28 and rank on a scale of 1-5: resolved without sequelae = 1, resolved with sequelae = 2, AE still present at study end/discontinuation = 3, subject died = 4, outcome unknown = 5.

**Date event resolved** = You are asked to complete this on Day 28 in case an event fluctuates in intensity (severity) during the course of follow-up. At the final visit, review the data on the CRF with the patient to determine the date that the event resolved. If they cannot remember the exact date, choose the date that is halfway between the date the event was last recorded as abnormal and the date that the event was recorded as normal. If a patient is still experiencing an AE on Day 28, consider consulting the Kampala core facility staff to discuss the plan for further follow-up vs. discharge from the study. Patients who are still experiencing an AE on Day 28 will be managed according to the following guidelines: a) if the AE is improving and is < grade 1 (mild), the patient will be discharged from the study and the date of resolution for the AE will be recorded as Day 28; b) if the AE is stable or worsening, the patient will continued to be followed until the AE resolves or improves.

**Management of serious adverse events**: Study coordinators in Kampala will be notified within 24 hours of any serious adverse event. A serious adverse event is one that is:

- Fatal
- Life-threatening
- Results in or prolongs hospitalization
- Requires medical or surgical interventions to prevent a serious outcome
- Results in significant or persistent disability or capacity

Any death that occurs during the study will be considered a serious adverse event, regardless of whether an AE had previously been reported for the patient. If indicated, the study patient will be transferred to the nearest district hospital for appropriate care, including any necessary laboratory investigations.

**Length of follow-up for adverse events (See Appendix 14)**

1. **AEs presenting during the 28-day follow-up period** - If a patient with a previously reported AE is still experiencing the AE on Day 28, or if a patient presents with a new AE on Day 28, the Kampala core facility staff should be notified to discuss the plan for further follow-up vs. discharge from the study. Patients with ongoing AEs on Day 28 will be managed according to the following guidelines:
   1. If the AE is “old” (previously reported on Days 0-27) and is < grade 1 (mild), the patient will be managed according to good medical practice and will be discharged from the study. The date of resolution for the AE will be recorded as Day 28.
   2. If the AE is “old” (previously reported on Days 0-27) and is > grade 1, the patient will be followed until the AE resolves, improves, or stabilizes.
   3. If the AE is new, the AE will be reported and the patient will be followed until the AE resolves, improves, or stabilizes.
2. **AEs presenting after Day 28** – If a patient presents with a new AE during a follow-up visit after Day 28, the new AE will be managed according to the following guidelines:
   1. If the AE is serious, and the relationship of the event to study medications is “NONE” or “UNLIKELY”, the patient will be managed according to good medical practice, but the AE will not be reported or followed.
   2. If the AE is serious, and the relationship of the event to study medications is “POSSIBLY”, “PROBABLY” or “DEFINITELY”, the AE will be reported and the patient will be followed until the AE resolves, improves, or stabilizes.
   3. If the AE is not serious, the patient will be managed according to good medical practice, but the AE will not be reported or followed.
3. **Serious AEs** – Any patient who experiences a serious AE should be followed until the AE resolves or improves (< grade 1). Please note that if a patient develops evidence of severe malaria or danger signs, and requires treatment with parenteral quinine +/- hospitalization, the symptoms / signs / laboratory abnormalities leading to the diagnosis of severe malaria will be classified as serious AEs. Although formal study follow-up is typically terminated when a patient is classified as a treatment failure, any patient with severe malaria / danger signs should be followed up to ensure their serious AE has resolved / improved, and data should be recorded on the Serious Adverse Event – Follow-up Form.
4. **AEs in patients classified as treatment failures** – Formal study follow-up ends when a patient is classified as a treatment failure (ETF or LTF), and patients should be treated with quinine and managed according to good medical practice. Additional follow-up for AEs in patients classified as treatment failures, is not typically indicated, unless the AE is serious, or is felt to be probably or definitely related to the study medications. The Kampala core facility staff should be contacted with any questions about follow-up of AEs in patients with treatment failure.

**Appendix 11a**

| **SERIOUS adverse even form – initial report** | | |
| --- | --- | --- |
| **1. Study U**|___|___|___|___|___|  **Number:** | **2. Day 0 Date:** |___|___|/|___|___|/|___|___|  ***day month year*** | **3. Treatment**  **Number:**  |___|___|___| |

| Event description:______________________________________________________________________________  *(symptom, sign, or laboratory abnormality)* | | | | |
| --- | --- | --- | --- | --- |
| Date of event onset:  |___|___|/|___|___|/|___|___|___|___|  day month year | | Date event reported:  |___|___|/|___|___|/|___|___|___|___|  day month year | | Indicate reason for serious AE:   Fatal   Life-threatening   Resulted in significant /  persistent disability or  incapacity   Resulted in hospitalization   Prolonged hospitalization   Required medical / surgical  intervention to prevent serious  outcome   Other:____________________ |
| Maximum event severity:   Moderate   Severe   Life-threatening | | Maximum relationship to study drugs:   None   Unlikely   Possible   Probable   Definite | |  |
| Was the event unexpected?  ____ Yes ____No | |  |  |  |
| Clinical history:  ________________________________________  ________________________________________  ________________________________________  ________________________________________  ________________________________________  ________________________________________  ________________________________________  ________________________________________  ________________________________________  ________________________________________  ________________________________________  ________________________________________  ________________________________________  ________________________________________  ________________________________________  ________________________________________  ________________________________________  ________________________________________  ________________________________________  ________________________________________  ________________________________________  ________________________________________ | | | Relevant past medical history:  ________________________________________  ________________________________________  ________________________________________  ________________________________________  ________________________________________ | |
|  |  |  | Concomitant medications:  1.__________________________________________  2.__________________________________________  3.__________________________________________  4.__________________________________________  5.__________________________________________ | |
|  |  |  | Action taken: (tick all that apply)   No change in current management   Study medication discontinued   Specific treatment given   Patient hospitalized   Laboratory tests obtained   Other:____________________   Other:____________________ | |
| Date form completed:  |___|___|/|___|___|/|___|___|___|___|  *day month year* | Investigator’s name (printed): ________________________________  Investigator’s signature: ______________________________________ | | | |

**Appendix 11b**

| **SERIOUS adverse even form – follow-up report** | | |
| --- | --- | --- |
| **1. Study U**|___|___|___|___|___|  **Number:** | **2. Day 0 Date:** |___|___|/|___|___|/|___|___|  ***day month year*** | **3. Treatment**  **Number:**  |___|___|___| |

| Date of follow-up: |___|___|/|___|___|/|___|___|  *day month year* | | | Study Day: | Temp: | |
| --- | --- | --- | --- | --- | --- |
| Progress Note:  _______________________________________________________  _______________________________________________________  _______________________________________________________  _______________________________________________________  _______________________________________________________  _______________________________________________________  _______________________________________________________ | | | Laboratory results / Other comments: | | |
| Date of follow-up: |___|___|/|___|___|/|___|___|  *day month year* | | | Study Day: | | Temp: |
| Progress Note:  _______________________________________________________  _______________________________________________________  _______________________________________________________  _______________________________________________________  _______________________________________________________  _______________________________________________________  _______________________________________________________ | | | Laboratory results / Other comments: | | |
| Date of follow-up: |___|___|/|___|___|/|___|___|  *day month year* | | | Study Day: | | Temp: |
| Progress Note:  _______________________________________________________  _______________________________________________________  _______________________________________________________  _______________________________________________________  _______________________________________________________ | | | Laboratory results / Other comments: | | |
| Date of follow-up: |___|___|/|___|___|/|___|___|  *day month year* | | | Study Day: | | Temp: |
| Progress Note:  _______________________________________________________  _______________________________________________________  _______________________________________________________  _______________________________________________________  _______________________________________________________ | | | Laboratory results / Other comments: | | |
| Outcome:  □ Resolved  □ Ongoing  □ Died | If resolved, date of resolution:  |___|___|/|___|___|/|___|___|___|___|  *day month year* | Investigator’s signature:  ________________________________  Date:________________________________ | | | |

###### APPENDIX 12. Expected Adverse Events for study medications.

| **Skin** | Skin rash  Pruritis  Depigmentation  Hair loss  Bluish-gray pigmentation of the fingernails, skin, hard palate  Erythema multiforme  Steven’s-Johnson Lyell’s syndrome |
| --- | --- |
| **GI** | Nausea  Vomiting  Diarrhea  Hepatitis  Feeling of fullness  Stomatitis |
| **Heme** | Leukopenia  Agranulocytosis  Aplastic anemia  Hemolytic anemia  Megaloblastic anemia  Thrombocytopenia  Purpura |
| **Eye** | Visual disturbances  Corneal deposits or opacities Retinal degeneration |
| **Neuro** | Peripheral neuropathy  Polyneuritis  Neurological and psychiatric changes  Convulsions  Psychosis |
| **Other** | Fever  Lethargy / fatigue  Headache  Allergic reactions  EKG changes  Cardiomyopathy  Pulmonary infiltrates |

****Expected AEs for CQ obtained from Avloclor package insert – – Cosmoquin package insert unavailable***

**Appendix 13. Criteria for Severe Malaria/Danger Signs**

**Severe Malaria**

- Unarousable coma *(if after convulsion, > 30 min)*
- Repeated convulsions *(> 2 within 24 h)*
  - Severe anemia *(Hb < 5.0 g/dL)*
  - Respiratory distress *(laboured breathing at rest)*
  - Jaundice *(yellow coloring of eyes)*

**Danger Signs**

- Recent convulsions *(1-2 within 24 h)*
- Altered consciousness  *(confusion, delerium, psychosis)*
- Lethargy
  - Unable to drink or breast feed
  - Vomiting everything
  - Unable to stand/sit due to weakness

**Appendix 14: UMSP Adverse Event Follow-up Guidelines**

UMSP Adverse Event Follow-up Guidelines

* Manage the patient according to “Good Medical Practice” and follow-up as clinically appropriate. From the standpoint of the AE, study follow-up may be ended and the patient can be discharged from the study.

***Appendix 15. Clinical Record forms***

| **UMSP clinical record form (1): Two-Arm Study (CQ/SP, AQ/SP)** | | | | | | | | | |
| --- | --- | --- | --- | --- | --- | --- | --- | --- | --- |
| **Patient**  **Initials:** | **1. Study Number: U**|___|___|___|___|___| | | **2. Day 0 Date:** |___|___|/|___|___|/|___|___|  ***day month year*** | | | | | **3. Treatment Number:** |___|___|___| | |
| **4. Age:_______years______months**  *(include months only if age < 5 years, else write “X”)* | | **5. Gender: _____M _____F** | | | **6. Weight** *(kg):* | **7. Known drug allergies: ___Yes ___No ___Unknown**  **If yes, describe___________________________________** | | | |
| List all medications taken within the last 2 weeks | | | | | | | | | |
| **Drug** *(if name unknown, list by letter – “Unknown Drug A”)* **(a)** | | | | **Dose** *(# of tablets/injections)* **(b)** | | | **Dates given (c)** | | **Duration (d)** |
| 8. | | | |  | | |  | |  |
| 9. | | | |  | | |  | |  |
| 10. | | | |  | | |  | |  |
| 11. | | | |  | | |  | |  |

| symptom record ***(Rank on scale of 0-4: absent = 0; mild = 1; moderate = 2; severe = 3, life-threatening = 4, N/A = unable to assess)*** | | | | | | | | | | |
| --- | --- | --- | --- | --- | --- | --- | --- | --- | --- | --- |
|  | **day 0 (a)** | **day 1 (b)** | **day 2 (c)** | **day 3 (d)** | **day 7 (e)** | **day 14 (f)** | **day 21 (g)** | **day 28 (h)** | **day ---- (i)** | **day ---- (j)** |
| **DATE** |  |  |  |  |  |  |  |  |  |  |
| 12. Subjective fever  in past 24h *(Y/N)* |  |  |  |  |  |  |  |  |  |  |
| 13. Weakness |  |  |  |  |  |  |  |  |  |  |
| 14. Muscle/joint aches* |  |  |  |  |  |  |  |  |  |  |
| 15. Headache* |  |  |  |  |  |  |  |  |  |  |
| 16. Anorexia |  |  |  |  |  |  |  |  |  |  |
| 17. Nausea* |  |  |  |  |  |  |  |  |  |  |
| 18. Vomiting |  |  |  |  |  |  |  |  |  |  |
| 19. Abdominal pain* |  |  |  |  |  |  |  |  |  |  |
| 20. Diarrhea |  |  |  |  |  |  |  |  |  |  |
| 21. Cough |  |  |  |  |  |  |  |  |  |  |
| 22. Pruritis |  |  |  |  |  |  |  |  |  |  |
| 25. “Flu” |  |  |  |  |  |  |  |  |  |  |
| 26. Convulsions. |  |  |  |  |  |  |  |  |  |  |
| 27. Other____________ |  |  |  |  |  |  |  |  |  |  |
| 28. Adverse event  reported† *(Y/N)* |  |  |  |  |  |  |  |  |  |  |
| Initials |  |  |  |  |  |  |  |  |  |  |

****Only assess in children > 3 years of age. For children < 3 and those unable to answer, enter N/A.***

***† Adverse event reported if symptom is new or worsening and grade is > 2. Notify Kampala core facility immediately of all serious adverse events.***

| **UMSP clinical record form (2): Two-Arm Study (CQ/SP, AQ/SP)** | | | |
| --- | --- | --- | --- |
| **Patient**  **Initials:** | **1. Study Number: U**|___|___|___|___|___| | **2. Day 0 Date:** |___|___|/|___|___|/|___|___|  ***day month year*** | **3. Treatment Number:** |___|___|___| |

| **physical exam record**  *(Rank on scale of 0-4: normal = 0; mild abnormality = 1; moderate = 2; severe = 3, life-threatening = 4, N/A = unable to assess)* | | | | | | | | | | |
| --- | --- | --- | --- | --- | --- | --- | --- | --- | --- | --- |
|  | **day 0 (a)** | **day 1 (b)** | **day 2 (c)** | **day 3 (d)** | **day 7 (e)** | **day 14 (f)** | **day 21 (g)** | **day 28 (h)** | **day ---- (i)** | **day ---- (j)** |
| **DATE** |  |  |  |  |  |  |  |  |  |  |
| 29. Temperature *(ºC)* |  |  |  |  |  |  |  |  |  |  |
| 30. Dehydration |  |  |  |  |  |  |  |  |  |  |
| 31. Facial edema |  |  |  |  |  |  |  |  |  |  |
| 32. Jaundice |  |  |  |  |  |  |  |  |  |  |
| 33. Chest |  |  |  |  |  |  |  |  |  |  |
| 34. Abdomen |  |  |  |  |  |  |  |  |  |  |
| 35. Skin |  |  |  |  |  |  |  |  |  |  |
| 41. Other__________ |  |  |  |  |  |  |  |  |  |  |
| 42. Other__________ |  |  |  |  |  |  |  |  |  |  |
| 43. Adverse event  reported† *(Y/N)* |  |  |  |  |  |  |  |  |  |  |
| ABNORMAL EXAM RECORD | | | | | | | | | | |
| If abnormality noted on physical exam, describe all physical findings for the abnormal exam |  |  |  |  |  |  |  |  |  |  |
| Initials |  |  |  |  |  |  |  |  |  |  |

**** Follow age-based guidelines: Tablet test – > 9 mo; Heel-toe – > 2 years; Romberg – > 4 years. Answer N/A for younger children and uncooperative patients.***

***† Adverse event reported if exam sign is new or worsening and grade is > 2. Notify Kampala core facility immediately of all serious adverse events.***

| **UMSP clinical record form (3): Two-Arm Study (CQ/SP, AQ/SP)** | | | |
| --- | --- | --- | --- |
| **Patient**  **Initials:** | **1. Study Number: U**|___|___|___|___|___| | **2. Day 0 Date:** |___|___|/|___|___|/|___|___|  ***day month year*** | **3. Treatment Number:** |___|___|___| |

| *LABORATORY RECORD* | | | | | | | | | | |
| --- | --- | --- | --- | --- | --- | --- | --- | --- | --- | --- |
|  | **day 0 (a)** | **day 1 (b)** | **day 2 (c)** | **day 3 (d)** | **day 7 (e)** | **day 14 (f)** | **day 21 (g)** | **day 28 (h)** | **day ---- (i)** | **day ---- (j)** |
| 44. Parasite density  *(asexual parasites/ul)* |  |  |  |  |  |  |  |  |  |  |
| 45. Species |  |  |  |  |  |  |  |  |  |  |
| 46. Gametocytes *(Y/N)* |  |  |  |  |  |  |  |  |  |  |
| 49. Hemoglobin*† *(g/dL)*  [grade] | [ ] |  |  |  |  |  |  | [ ] |  |  |
| Initials |  |  |  |  |  |  |  |  |  |  |

****(Grade on scale of 0-4: normal = 0; mild abnormality = 1; moderate = 2; severe = 3, life-threatening = 4)***

***† Adverse event reported if hemoglobin measured after Day 0 is grade > 2. Notify Kampala core facility immediately of all serious adverse events.***

| RECORD OF ADDITIONAL MEDICATION GIVEN DURING STUDY | | | | | |
| --- | --- | --- | --- | --- | --- |
| **Medication (a)** | **Indication (b)** | **Dose (c)** | **Duration (d)** | **Date started (e)** | **Study day (f)** |
| **60.** |  |  |  |  |  |
| **61.** |  |  |  |  |  |
| **62.** |  |  |  |  |  |
| **63.** |  |  |  |  |  |
| **64.** |  |  |  |  |  |
| **65.** |  |  |  |  |  |
| *66.* |  |  |  |  |  |
| **67.** |  |  |  |  |  |
| **68.** |  |  |  |  |  |
| **69.** |  |  |  |  |  |
| **70.** |  |  |  |  |  |

| **UMSP clinical record form (4): Two-Arm Study (CQ/SP, AQ/SP** | | | |
| --- | --- | --- | --- |
| **Patient**  **Initials:** | **1. Study Number: U**|___|___|___|___|___| | **2. Day 0 Date:** |___|___|/|___|___|/|___|___|  ***day month year*** | **3. Treatment Number:** |___|___|___| |

| complete efficacy outcome | |
| --- | --- |
|  ETF     LCF   LPF   ACPR     N/A (Tick appropriate Incomplete Efficacy Outcome **)** | If failed, **STUDY DAY** of clinical failure (0-28) __________  **Reason for Clinical Failure:**   Severe malaria/danger signs with parasitemia Days 0-3 🡪 Specify criteria _________   Severe AE requiring change in treatment Days 0-2 🡪 Specify criteria _____________   Day 2 parasite count > Day 0 count   Parasitemia on Day 3 with temperature > 37. 5   Day 3 parasite count > 25% Day 0 count   Severe malaria with parasitemia Days 4-28 🡪 Specify criteria __________________   Parasitemia on Days 4-28 with temperature > 37. 5 OR History of fever in past 24 hours. |

**Outcome Classification**

**ETF🡪** Assessed Days 0-3

**LCF🡪** Assessed Days 4-28 and previously not an ETF.

**LPF🡪** Assessed Day28

and previously not an ETF

or LCF.

**ACPR🡪** Assessed Day28 and previously not an ETF, LCF or LPF.

**For exact definitions refer to:**

- Study Poster in Clinic
- Field Manual Appendix 3
- SOP # DATA3-003 CRF

| INCOMPLETE EFFICACY OUTCOME |
| --- |
|  **Excluded** - If yes, reason for exclusion: (Must tick one reason below)   Other antimalarial use: if yes, describe__________________________   Withdrew informed consent   Concomitant febrile illness: if yes, diagnosis_____________________   Lost - If yes, last Study day examined: _____________   Error made during follow-up that prevented outcome classification: ______________________________ |

| **UMSP clinical record form (1): Three-Arm Study (CQ/SP, AQ/SP, AQ/AS)** | | | | | | | | | |
| --- | --- | --- | --- | --- | --- | --- | --- | --- | --- |
| **Patient**  **Initials:** | **1. Study Number: U**|___|___|___|___|___| | | **2. Day 0 Date:** |___|___|/|___|___|/|___|___|  ***day month year*** | | | | | **3. Treatment Number:** |___|___|___| | |
| **4. Age:_______years______months**  *(include months only if age < 5 years, else write “X”)* | | **5. Gender: _____M _____F** | | | **6. Weight** *(kg):* | **7. Known drug allergies: ___Yes ___No ___Unknown**  **If yes, describe___________________________________** | | | |
| **List all medications taken within the last 2 weeks** | | | | | | | | | |
| **Drug** *(if name unknown, list by letter – “Unknown Drug A”)* **(a)** | | | | **Dose** *(# of tablets/injections)* **(b)** | | | **Dates given (c)** | | **Duration (d)** |
| 8. | | | |  | | |  | |  |
| 9. | | | |  | | |  | |  |
| 10. | | | |  | | |  | |  |
| 11. | | | |  | | |  | |  |

| **symptom record**  ***(Rank on scale of 0-4: absent = 0; mild = 1; moderate = 2; severe = 3, life-threatening = 4, N/A = unable to assess)*** | | | | | | | | | | |
| --- | --- | --- | --- | --- | --- | --- | --- | --- | --- | --- |
|  | **day 0 (a)** | **day 1 (b)** | **day 2 (c)** | **day 3 (d)** | **day 7 (e)** | **day 14 (f)** | **day 21 (g)** | **day 28 (h)** | **day ---- (i)** | **day ---- (j)** |
| **DATE** |  |  |  |  |  |  |  |  |  |  |
| 12. Subjective fever  in past 24h *(Y/N)* |  |  |  |  |  |  |  |  |  |  |
| 13. Weakness |  |  |  |  |  |  |  |  |  |  |
| 14. Muscle/joint aches* |  |  |  |  |  |  |  |  |  |  |
| 15. Headache* |  |  |  |  |  |  |  |  |  |  |
| 16. Anorexia |  |  |  |  |  |  |  |  |  |  |
| 17. Nausea* |  |  |  |  |  |  |  |  |  |  |
| 18. Vomiting |  |  |  |  |  |  |  |  |  |  |
| 19. Abdominal pain* |  |  |  |  |  |  |  |  |  |  |
| 20. Diarrhea |  |  |  |  |  |  |  |  |  |  |
| 21. Cough |  |  |  |  |  |  |  |  |  |  |
| 22. Pruritis |  |  |  |  |  |  |  |  |  |  |
| 23. Tinnutus* |  |  |  |  |  |  |  |  |  |  |
| 24. Behavioural changes |  |  |  |  |  |  |  |  |  |  |
| 25. “Flu” |  |  |  |  |  |  |  |  |  |  |
| 26. Convulsions |  |  |  |  |  |  |  |  |  |  |
| 27. Other____________ |  |  |  |  |  |  |  |  |  |  |
| 28. Adverse event  reported† *(Y/N)* |  |  |  |  |  |  |  |  |  |  |
| Initials |  |  |  |  |  |  |  |  |  |  |

****Only assess in children > 3 years of age. For children < 3 and those unable to answer, enter N/A.***

***† Adverse event reported if symptom is new or worsening and grade is >*** 2. Notify Kampala core facility immediately of all serious adverse events.

| **UMSP clinical record form (2): Three-Arm Study (CQ/SP, AQ/SP, AQ/AS)** | | | |
| --- | --- | --- | --- |
| **Patient**  **Initials:** | **1. Study Number: U**|___|___|___|___|___| | **2. Day 0 Date:** |___|___|/|___|___|/|___|___|  ***day month year*** | **3. Treatment Number:** |___|___|___| |

| **physical exam record**  *(Rank on scale of 0-4: normal = 0; mild abnormality = 1; moderate = 2; severe = 3, life-threatening = 4, N/A = unable to assess)* | | | | | | | | | | |
| --- | --- | --- | --- | --- | --- | --- | --- | --- | --- | --- |
|  | **day 0 (a)** | **day 1 (b)** | **day 2 (c)** | **day 3 (d)** | **day 7 (e)** | **day 14 (f)** | **day 21 (g)** | **day 28 (h)** | **day ---- (i)** | **day ---- (j)** |
| **DATE** |  |  |  |  |  |  |  |  |  |  |
| 29. Temperature *(ºC)* |  |  |  |  |  |  |  |  |  |  |
| 30. Dehydration |  |  |  |  |  |  |  |  |  |  |
| 31. Facial edema |  |  |  |  |  |  |  |  |  |  |
| 32. Jaundice |  |  |  |  |  |  |  |  |  |  |
| 33. Chest |  |  |  |  |  |  |  |  |  |  |
| 34. Abdomen |  |  |  |  |  |  |  |  |  |  |
| 35. Skin |  |  |  |  |  |  |  |  |  |  |
| 36. Hearing |  |  |  |  |  |  |  |  |  |  |
| 37. Nystagmus |  |  |  |  |  |  |  |  |  |  |
| 38. Tablet test* |  |  |  |  |  |  |  |  |  |  |
| 39. Heel-toe* |  |  |  |  |  |  |  |  |  |  |
| 40. Romberg* |  |  |  |  |  |  |  |  |  |  |
| 41. Other__________ |  |  |  |  |  |  |  |  |  |  |
| 42. Other__________ |  |  |  |  |  |  |  |  |  |  |
| 43. Adverse event  reported† *(Y/N)* |  |  |  |  |  |  |  |  |  |  |
| ABNORMAL EXAM RECORD | | | | | | | | | | |
| If abnormality noted on physical exam, describe all physical findings for the abnormal exam |  |  |  |  |  |  |  |  |  |  |
| Initials |  |  |  |  |  |  |  |  |  |  |

**** Follow age-based guidelines: Tablet test – > 9 mo; Heel-toe – > 2 years; Romberg – > 4 years. Answer N/A for younger children and uncooperative patients.***

***† Adverse event reported if exam sign is new or worsening and grade is > 2. Notify Kampala core facility immediately of all serious adverse events.***

| **UMSP clinical record form (3): Three-Arm Study (CQ/SP, AQ/SP, AQ/AS)** | | | |
| --- | --- | --- | --- |
| **Patient**  **Initials:** | **1. Study Number: U**|___|___|___|___|___| | **2. Day 0 Date:** |___|___|/|___|___|/|___|___|  ***day month year*** | **3. Treatment Number:** |___|___|___| |

| *LABORATORY RECORD* | | | | | | | | | | |
| --- | --- | --- | --- | --- | --- | --- | --- | --- | --- | --- |
|  | **day 0 (a)** | **day 1 (b)** | **day 2 (c)** | **day 3 (d)** | **day 7 (e)** | **day 14 (f)** | **day 21 (g)** | **day 28 (h)** | **day ---- (i)** | **day ---- (j)** |
| 44. Parasite density  *(asexual parasites/ul)* |  |  |  |  |  |  |  |  |  |  |
| 45. Species |  |  |  |  |  |  |  |  |  |  |
| 46. Gametocytes *(Y/N)* |  |  |  |  |  |  |  |  |  |  |
| 49. Hemoglobin*† *(g/dL)*  [grade] | [ ] |  |  |  |  |  |  | [ ] |  |  |
| Initials |  |  |  |  |  |  |  |  |  |  |

****(Grade on scale of 0-4: normal = 0; mild abnormality = 1; moderate = 2; severe = 3, life-threatening = 4)***

***† Adverse event reported if hemoglobin measured after Day 0 is grade > 2. Notify Kampala core facility immediately of all serious adverse events.***

| RECORD OF ADDITIONAL MEDICATION GIVEN DURING STUDY | | | | | |
| --- | --- | --- | --- | --- | --- |
| **Medication (a)** | **Indication (b)** | **Dose (c)** | **Duration (d)** | **Date started (e)** | **Study day (f)** |
| **60.** |  |  |  |  |  |
| **61.** |  |  |  |  |  |
| **62.** |  |  |  |  |  |
| **63.** |  |  |  |  |  |
| **64.** |  |  |  |  |  |
| **65.** |  |  |  |  |  |
| *66.* |  |  |  |  |  |
| **67.** |  |  |  |  |  |
| **68.** |  |  |  |  |  |
| **69.** |  |  |  |  |  |
| **70.** |  |  |  |  |  |

| **UMSP clinical record form (4): Three-Arm Study (CQ/SP, AQ/SP, AQ/AS)** | | | |
| --- | --- | --- | --- |
| **Patient**  **Initials:** | **1. Study Number: U**|___|___|___|___|___| | **2. Day 0 Date:** |___|___|/|___|___|/|___|___|  ***day month year*** | **3. Treatment Number:** |___|___|___| |

| complete efficacy outcome | |
| --- | --- |
|  ETF     LCF   LPF   ACPR     N/A (Tick appropriate Incomplete Efficacy Outcome **)** | If failed, **STUDY DAY** of clinical failure (0-28) __________  **Reason for Clinical Failure:**   Severe malaria/danger signs with parasitemia Days 0-3 🡪 Specify criteria _________   Severe AE requiring change in treatment Days 0-2 🡪 Specify criteria _____________   Day 2 parasite count > Day 0 count   Parasitemia on Day 3 with temperature > 37. 5   Day 3 parasite count > 25% Day 0 count   Severe malaria with parasitemia Days 4-28 🡪 Specify criteria __________________   Parasitemia on Days 4-28 with temperature > 37. 5 OR History of fever in past 24 hours. |

**Outcome Classification**

**ETF🡪** Assessed Days 0-3

**LCF🡪** Assessed Days 4-28 and previously not an ETF.

**LPF🡪** Assessed Day28

and previously not an ETF

or LCF.

**ACPR🡪** Assessed Day28 and previously not an ETF, LCF or LPF.

**For exact definitions refer to:**

- Study Poster in Clinic
- Field Manual Appendix 3
- SOP # DATA3-003 CRF

| INCOMPLETE EFFICACY OUTCOME |
| --- |
|  **Excluded** - If yes, reason for exclusion: (Must tick one reason below)   Other antimalarial use: if yes, describe__________________________   Withdrew informed consent   Concomitant febrile illness: if yes, diagnosis_____________________   Lost - If yes, last Study day examined: _____________   Error made during follow-up that prevented outcome classification: ______________________________ |

Appendix 16. UMSP Adverse Event Record Form

| **adverse event record form** | | | |
| --- | --- | --- | --- |
| **Patient**  **Initials:** | **1. Study Number: U**|___|___|___||___||___| | **2. Day 0 Date:** |___|___|/|___|___|/|___|___|  ***day month year*** | **3. Treatment Number:** |___|___|___| |

|  | ***Complete on day first reported*** | | | ***Complete on day first reported and update as needed*** | | | ***Complete on final day*** | | |
| --- | --- | --- | --- | --- | --- | --- | --- | --- | --- |
| **Event description (a)** | **Date of event onset (b)** | **Date event reported**  **(c)** | **Initials of person reporting** | **Maximum severity* (d)** | **Maximum relationship† (e)** | **Serious? ‡ *(Y/N)***  **(f)** | **Episodic?**  ***(Y/N)***  **(g)** | **Outcome †† (h)** | **Date event resolved‡‡ (i)** |
| **80.** |  |  |  |  |  |  |  |  |  |
| **81.** |  |  |  |  |  |  |  |  |  |
| **82.** |  |  |  |  |  |  |  |  |  |
| **83.** |  |  |  |  |  |  |  |  |  |
| **84.** |  |  |  |  |  |  |  |  |  |
| **85.** |  |  |  |  |  |  |  |  |  |
| **86.** |  |  |  |  |  |  |  |  |  |
| **87.** |  |  |  |  |  |  |  |  |  |
| **88.** |  |  |  |  |  |  |  |  |  |
| **89.** |  |  |  |  |  |  |  |  |  |
| **90.** |  |  |  |  |  |  |  |  |  |
| **91.** |  |  |  |  |  |  |  |  |  |
| **92.** |  |  |  |  |  |  |  |  |  |
| **93.** |  |  |  |  |  |  |  |  |  |
| **94.** |  |  |  |  |  |  |  |  |  |

*** d) Severity:** *Rank on scale of 1-4: mild = 1; moderate = 2; severe = 3, life-threatening = 4*

**† e) Relationship:** *Rank on scale of 0-4: none = 0; unlikely = 1; possible = 2; probable = 3; definite = 4*

**‡ f) Serious:** Criteria for serious AE: fatal, life-threatening, results in or prolongs hospitalization, results in significant or persistent disability or capacity requires medical / surgical intervention to prevent serious outcome. **If serious, report to Kampala core facility staff immediately. They will assist with patient management, assist with completion of serious AE forms, and report the AE if necessary.**

**††h) Outcome:** *Rank on scale of 1-5: resolved without sequelae = 1; resolved with sequelae = 2; AE still present at study end/discontinuation, but improving = 3; subject died = 4; unknown = 5*

**‡‡ i) Date event resolved:** *Complete on Day 28 – If AE still ongoing at end of follow-up, indicate in question (h).*
